# Epidemiological Evidence for Work Load as a Risk Factor for Osteoarthritis of the Hip: A Systematic Review

**DOI:** 10.1371/journal.pone.0031521

**Published:** 2012-02-14

**Authors:** Sandra I. Sulsky, Laura Carlton, Frank Bochmann, Rolf Ellegast, Ulrich Glitsch, Bernd Hartmann, Dirk Pallapies, D. Seidel, Yi Sun

**Affiliations:** 1 ENVIRON International Corporation, Amherst, Massachusetts, United States of America; 2 Institut für Arbeitsschutz der Deutschen Gesetzlichen Unfallversicherung (IFA), Sankt Augustin, Germany; 3 Berufsgenossenschaft der Bauwirtschaft (BG BAU), Arbeitsmedizinisch-Sicherheitstechnischer Dienst, Berlin, Germany; 4 Institut für Prävention und Arbeitsmedizin der Deutschen Gesetzlichen Unfallversicherung (IPA), Bochum, Germany; University of Southampton, United Kingdom

## Abstract

**Objective:**

Osteoarthritis of the hip (OA) is a common degenerative disorder of the joint cartilage that presents a major public health problem worldwide. While intrinsic risk factors (e.g, body mass and morphology) have been identified, external risk factors are not well understood. In this systematic review, the evidence for workload as a risk factor for hip OA is summarized and used to derive recommendations for prevention and further research.

**Methods:**

Epidemiological studies on workload or occupation and osteoarthritis of the hip were identified through database and bibliography searches. Using pre-defined quality criteria, 30 studies were selected for critical evaluation; six of these provided quantitative exposure data.

**Results:**

Study results were too heterogeneous to develop pooled risk estimates by specific work activities. The weight of evidence favors a graded association between long-term exposure to heavy lifting and risk of hip OA. Long-term exposure to standing at work might also increase the risk of hip OA.

**Conclusions:**

It is not possible to estimate a quantitative dose-response relationship between workload and hip OA using existing data, but there is enough evidence available to identify job-related heavy lifting and standing as hazards, and thus to begin developing recommendations for preventing hip OA by limiting the amount and duration of these activities. Future research to identify specific risk factors for work-related hip OA should focus on implementing rigorous study methods with quantitative exposure measures and objective diagnostic criteria.

## Introduction

Osteoarthritis or osteoarthrosis of the hip (hip OA) is a degenerative disorder of the joint cartilage and its underlying bone that can affect one or both hip joints [1]. It is a common joint disease in the elderly, is recognized as a major cause of pain, disability, and social expenditure, and presents a major public health problem throughout the world [2]–[5].

The diagnosis of hip OA is based on clinical criteria consisting of hip pain and impaired inner rotation in combination with radiographically confirmed joint degeneration [6]. Hip pain alone is not a sufficient indicator for OA because the majority of people with hip complaints do not show clinical or radiological evidence of osteoarthrosis, and not all people with radiographically confirmed hip OA experience pain [7]. Several systems for scoring morphological changes in hip joints have been developed and used over time, and they are more or less comparable. However, there is no gold standard method of scoring the radiographic evidence of hip OA. The first standardized system was proposed by Kellgren and Lawrence (K&L-score) in 1957 [8]. Their criteria were later accepted as a diagnostic method by the World Health Organization [9]. Revisions have attempted to address questions about the validity of the original scoring system, mainly related to the relative importance of the presence of osteophytes for defining OA (K&L-score grade 2 and above). Some believe that osteophytes are a natural phenomenon of age-related bone and joint remodeling, and thus should not contribute to the diagnosis of pathology [10]. Others suggest that the presence of osteophytes is the most specific criterion leading to OA diagnosis, and are as sensitive a criterion as joint space narrowing, especially for hip OA [11]. At least 10 other radiographic scoring systems for hip OA have been developed since the early 1980s [12]. These systems address nearly the same radiographic features, but weight their relative importance differently. Because the majority of persons with radiographic evidence of hip OA are symptom-free, alternative case definitions have been proposed. For example, epidemiological studies may identify OA cases on the basis of radiological findings, symptoms, or both. The primary consideration should be to develop consistent and reproducible methods for identifying cases.

Clinical and epidemiological studies of hip OA have investigated factors that correlated with the occurrence of symmetric OA including genetic predisposition [13], [14];arthritis of knee and finger joints, particularly in women [14]–[16]; malformations of hip, hip dysplasia or femoracetabular impingements [17]; demographic factors, such as race or gender [14], [18]; systemic factors, such as obesity or metabolic disorders [14], [18]; participation in certain sports, such as long distance running [19]; and occupational factors such as farming or heavy physical work load [18]. The peak incidence of hip OA is typically between ages 70 and 79 years [14]. Among women, the incidence rate per 100,000 person-years increases from approximately 50 in those aged 50–59 to approximately 600 in those aged 70–79; in men, incidence increases from approximately 30 in those aged 50–59 to approximately 430 per 100,000 person-years in those aged 70–79 [14]. Incidence and prevalence rates of hip OA vary across the world, but are generally high, and expected to increase due to demographic changes, in Western countries. The age standardized incidence rate for hip OA has been estimated as 53.3 per 100,000 for women and 38.1 per 100,000 for men in the defined WHO region EURO A of 26 European countries [20]. Prevalence rates for the same region were estimated at 577 per 100,000 for women and 413 per 100,000 for men. In Germany, about 8% of all orthopedic treatments and 2% of all early retirements were due to hip OA in 2002 [21].

The association between hip OA and heavy physical work load has been reported consistently in the scientific literature, but the size of the effect estimates and the specific occupational activities reported to increase risk vary between studies. Eleven systematic reviews published between January 1999 and March 2010 evaluated the association between occupational activities and/or work load and the risk of hip OA (Tables S1 and S2). The evidence for an effect of long-term physical work load or strain on the risk of hip OA was generally characterized as moderate or strong [22]–[28], or described as showing a positive or consistent association [29]–[31]. However, D'Souza found the methodological limitations of the underlying studies substantial enough to characterize the relationship with work load as only “suggestive” [32]. Lifting was identified as a specific risk factor for hip OA by several authors, with a few also characterizing risk associated by weight and frequency [24]–[26], [28], [31]. Other occupational activities considered to be among the characteristics of physically demanding work that increase the risk of hip OA included stair climbing, kneeling, and walking [25], [26], [30]. Two authors, however, judged the evidence for these associations with hip OA to be weak, inconsistent, and/or inadequate to reach a conclusion [23], [24], [28]. The lack of quantitative exposure data and/or reliance on cross-sectional or case-control study designs were consistently identified as weaknesses in the existing literature. Additionally, the variability across studies in methods and results has made developing specific preventive measures difficult, because of the uncertainty about key risk factors.

In all literature reviews and syntheses, quality differences between the underlying studies may play an important role in determining the size of the estimated effect. For example, the author of at least one review noted that studies with low quality scores tended to report higher effect estimates than studies with higher quality scores [25]. Most previous reviews have been qualitative, focusing on the authors' judgment regarding the association between physical work load and hip OA, rather than providing quantitative exposure-response relationships. As a result, the findings of these reviews provide only limited guidance for the development of preventive measures to reduce the incidence of work-related hip OA. The current review was undertaken with the goal of identifying valid, quantitative estimates of exposure to occupational activities that might increase the risk of hip OA, in order to develop specific work-place recommendations to prevent future cases. The analysis emphasizes studies with high quality designs and quantitative exposure data. Based on this analysis, we assessed the extent to which the current body of literature can inform the development of preventive measures for hip OA and occupational exposures. Possible prevention measures are discussed, along with recommendations on aspects of exposure assessment, diagnostic criteria and specific needs for future research.

## Methods

The authors identified studies from their personal libraries, and MEDLINE and EMBASE were searched to identify other studies of occupational work load and hip OA following previously recommended search strategies [33], [34]. Additional searches for literature reviews were done in MEDLINE, EMBASE and the Cochrane Occupational Health Field [35]. The bibliographies of key articles and the reviews were also checked for pertinent references not identified previously. An example of the multipart search strategy implemented in MEDLINE is as follows:

- Search (occupational diseases) OR (occupational risk)
- Search lifting OR carrying
- Search (hip joint endoprosthesis) OR (hip endoprosthesis) OR (hip joint prosthesis) OR (hip prosthesis) OR (“osteoarthritis, hip”[MeSH Terms] OR (“osteoarthritis”[All Fields] AND “hip”[All Fields]) OR “hip osteoarthritis”[All Fields] OR “coxarthroses”[All Fields])
- Search (“osteoarthritis, hip”[MeSH Terms] OR (“osteoarthritis”[All Fields] AND “hip”[All fields”] OR “hip osteoarthritis”[All Fields] OR “coxarthrosis”[All Fields]) OR (“osteoarthritis, hip”[MeSH Terms] OR (“osteoarthritis”[All Fields] AND “hip”[All Fields]) OR “hip osteoarthritis”[All Fields] OR “coxarthroses”[All Fields])

combine b OR c OR d = e

combine e AND a

Limits: Humans

**Figure 1:**
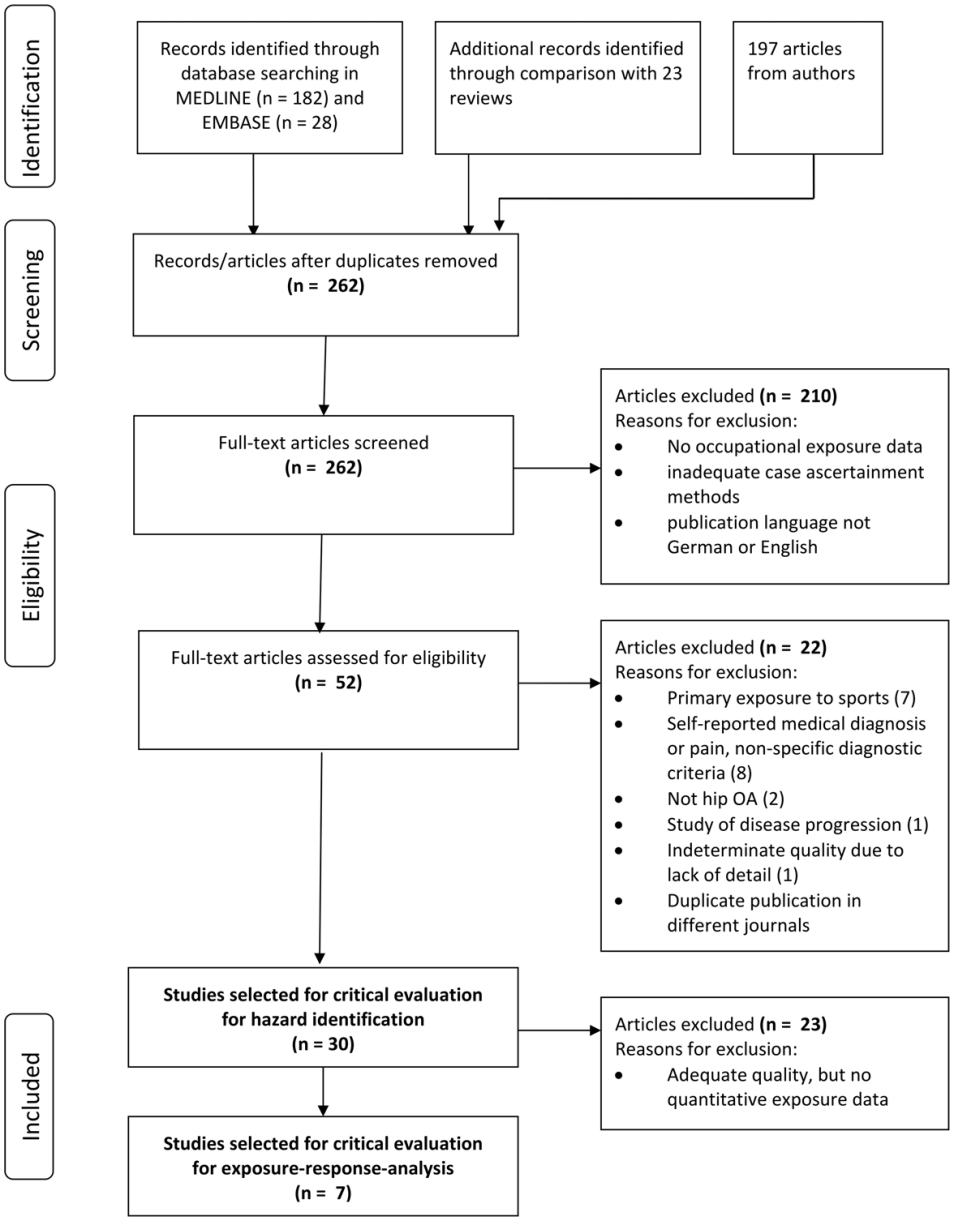
Flow diagram on identification of literature for critical evaluation.

After excluding duplicates, 262 original studies were screened to identify: 1, primary research; 2, studies that used radiographic evidence or total hip replacement surgery to diagnosis hip OA; and 3, studies that provided quantitative occupational exposure estimates. Studies that used quantitative exposure estimates but less rigorous case definitions, semi-quantitative exposure characterization (e.g., low, medium and high), or qualitative estimates of exposure intensity (e.g., “much” standing) were considered lesser quality, but included for their potential to contribute to the weight of evidence (Figure 1).

The majority of the initial screening and categorization of studies was performed by one staff person. One epidemiologist (LC) reviewed a random, 10% sample of all articles identified through database searches and other sources to assess the accuracy of the initial quality assessment, and a further 10% of the articles excluded at initial screening or classified in one of the “questionable quality” categories were re-screened. Among all articles rescreened for quality assurance, only one was found to have been excluded instead of classified as “questionable quality”; this study was later excluded for focusing on prognosis instead of incidence of hip OA.

## Results

Fifty-two of the 262 studies met preliminary inclusion criteria (Figure 1). After full text review by two epidemiologists (SS and LC), we implemented the following additional exclusions:

- Exposures were related to recreational or professional sports activity [19], [36]–[41];
- Cases were ascertained by self-reported prior hip OA diagnosis [42]–[45] or self-reported hip pain [46];
- Outcome was rheumatoid arthritis [47] or hand osteoarthritis [15], not hip OA;
- Outcome was progression from mild to severe hip OA, not development of hip OA [48];
- The data were duplicated in another publication [49]–[51];
- Joint spaces were measured, but criteria for diagnosing hip OA were not specified [52]–[54]; and
- Inadequate information was provided in the publication to allow a fair quality assessment [55].

We rated each of the 30 studies remaining for quality of their case ascertainment (Table 1) and exposure assessment methods (Table 2). Quality criteria and weighting of quality characteristics were developed and agreed upon prior to commencing the review (Table 3). The studies were divided between two epidemiologists (SS and LC) for quality assessment; a sample of studies was cross-reviewed to ensure agreement in quality assessments. Any discrepancies or questions were discussed until agreement was reached. The study attributes contributing the most weight to the quality assessments were: study design and methodology, likelihood of bias, and control for confounding (i.e., methodological threats to the validity of the reported findings). Characteristics most indicative of high quality study design included timing of exposure assessment (information gathered prior to onset of disease was considered more reliable); source and comparability of case and reference populations; and type of study, where cohort, case-control and cross-sectional designs were considered strongest to weakest, respectively. We judged the likelihood of selection bias, recall bias, and misclassification based on the description of the study methods, with special attention to the data collection methods and the sources and participation rates of case or exposed and reference populations. We looked specifically for information on the following potential confounders: age, gender, some measure of weight (e.g., body mass index, obesity), smoking, and non-occupational exposures. The clarity of the text describing study methods and results also factored in to our quality assessment, such that studies whose methods were unclear or that did not provide specific results received lower quality scores.

**Table 1:** Definition of level of evidence for diagnostic evaluation of studies on osteoarthritis of the hip.

Diagnosis Criteria	Score*: Case ascertainment
Medical history/questionnaire: hip pain without clinical check	1
Hip pain and clinical reduction of movement without radiographic featuresorRadiographic features without clinical examination without THR	2
Hip pain with clinical reduction of movement and clearly defined radiographic features (joint space narrowing or K&L-score grade 2 and above or comparable criteria)orDiagnostic with indication for total hip replacement (THR)	3

*Score 1 = low quality; score 3 = high quality.

**Table 2:** Definition of quality for exposure assessment of studies on osteoarthritis of the hip (score 1–5).

Exposure Assessment	Score*: Exposure assessment
Profession, job title, classification of occupation	1
Qualitative specification of exposure in different work activities (lifting, climbing stairs, sitting)	2
Quantitative specification of exposure in different work activities/physical strains with information on intensity (e.g. load weight steps) and duration	3
Quantitative specification of exposure (as above) with additional plausibility check (e.g. information on daily work output or special controls through video analysis)	4
Direct measurement or biomechanical model calculation of hip joint strain	5

*Score 1 = low quality; score 5 = high quality.

**Table 3:** Study attributes and their contribution to assessment of quality.

Study Attribute	Quality criteria
Study objectives	Clearly statedRelevant to research questions
Study methods	Adequately describedAppropriate for objectivesMinimize selection and information biasReasonable statistical power
Outcome measurement	Well-defined, reasonably specificAccurate measurement or diagnosisProper time frame for risk of outcome
Exposure measurement	Individual level, not group level dataDirect quantitative measurementsAccounts for changes over time
Control of confounding	Known risk factors considered and measuredReasonable analysis method(s) used (stratification, multivariate statistical models)

Summary quality assessments for all 30 studies are provided in Table S3. Details, including key elements of study design and specific results, are provided in Table S4 and discussed in the Appendix S1. After considering the exposure and outcome ascertainment scores and the study quality, six of the 30 studies were found to include quantitative exposure data. Of the 24 studies that could not be used for an exposure-response analysis:

- Four employed quantitative occupational exposure assessments, but used less rigorous case identification methods, were assessed as prohibitively low quality, or expressed exposures in non-generalizable (farming-specific) metrics;
- Seven employed semi-quantitative or qualitative estimates of exposure intensity with varying adequacy of case definition; and
- Thirteen employed only qualitative occupational exposure assessments with varying adequacy of case definition.

Results from these 24 studies nevertheless contribute to the weight of evidence regarding the presence/absence of an association between occupational physical demands and risk of hip OA. Nearly all of the studies reported higher prevalence of hip OA among the groups identified as exposed to higher levels of occupational physical activity or workload compared to others. Exceptions were Cvijectic et al. [56], Jacobsen et al. [53], and van Dijk et al. [57]. These authors did not detect statistically significant associations between risk of radiographically or clinically determined hip OA and either occupational work load or duration of exposure to work load categorized by level of intensity. Each of these studies was hampered by methodological problems, however, including potential information bias and misclassification of exposure and outcome. If in operation, any or all of these limitations would have reduced the magnitude of any true association between work load and hip OA, if one existed. Jacobsen et al. [53] was additionally difficult to interpret due to the authors' failure to report specific study results. Study details are provided in the Appendix S1 and Tables S3 and S4.

Cohort studies are generally considered the study design least prone to bias. Each of the five cohort studies included in this review [58]–[62] are briefly described, below, with more details provided in the Appendix S1 and Table S3 and S4. All five studies were limited by the authors' use of qualitative or semi-quantitative exposure metrics.

Flugsrud et al. (case ascertainment score: 3; exposure assessment score: 2) [58] matched exposure data from a large prospective cohort study with outcome data from a national THR registry to examine risk factors for hip OA in Norway. A total of 50,034 participants were eventually enrolled, including 672 participants who underwent a first THR for primary hip OA (cases). By using THR surgery to define cases, some participants with hip OA who had not undergone THR may be misclassified as non-cases, potentially biasing risk estimates towards the null.

At baseline, participants classified their work during the past year as sedentary or requiring moderate, intermediate, or intensive physical activity. There were 6–12 years between baseline assessments and start of follow-up, and the average duration of follow-up was nine years. Analyses were performed using Cox proportional hazards regression, adjusting for age at screening, body mass index, sex, body weight, marital status, smoking status, and physical activity during leisure time. Compared to those with self-rated sedentary jobs, risk of THR for hip OA was higher for those in higher categories self-rated work intensity among both men and women. After adjusting for all variables described above, the hazard ratio (HR) for men was 1.5 (95% CI: 1.0, 2.2) for moderate work, 1.7 (95% CI: 1.1, 2.4) for intermediate work, and 2.1 (95% CI: 1.5, 3.0) for intensive work compared to sedentary work. Compared to sedentary workers, women had HRs of 1.1 (95% 0.8, 1.6) for work in the moderate category, 1.4 (95% CI: 0.9, 2.0) for work in the intermediate category, and 2.1 (95% CI: 1.3, 3.3) for intensive work activity compared to sedentary work. Tests for trend were statistically significant for men (p<0.0001) and women (p<0.0003). In contrast, after controlling for all other variables, risk was not associated with higher categories of intensity of leisure activity for either men or women.

Jarvholm et al. (case ascertainment score: 3; exposure assessment score: 1) [59] collected data at multiple time points and recorded information on potential confounders (smoking habits, body weight and height), but categorized exposure only on the basis of job title from the first examination and did not report the results of any analyses that assessed or controlled for confounding. The cohort comprised Swedish men who received physical examinations every three to five years between 1971 and 1992. Hip OA incidence from 1987 to 1998 was determined through record linkage with the Swedish Hospital Discharge Register. Cases were defined as workers receiving surgical treatment for primary OA in the hip, with traumatic, secondary OA, or secondary hip replacement cases excluded. Relative risks for construction workers compared to white collar workers were estimated by Mantel-Haenszel statistics. After all exclusions were applied, the analytic cohort included 204,741 men, of whom 9,136 were considered to be white-collar workers. A total of 1,260 cases of hip OA were identified during the follow-up period. Relative risk estimates for hip OA by construction job title ranged from 0.77 to 1.58 when compared to white-collar workers, with none statistically significant. As with the Flugsrud et al. study, the use of THR surgery to define cases would bias risk estimates towards the null if the comparison group included men with hip OA that had not yet been surgically treated.

Between 1978 and 1980, Juhakoski et al. (case ascertainment score: 2; exposure assessment score: 2) [60] invited a representative sample of 8,000 Finns ages 30 and over, identified from population registers, to participate in the Mini-Finland Health Examination Survey. Ninety percent (7,217 subjects) completed baseline questionnaires, interviews, and laboratory and joint function tests. Those who reported experiencing hip pain leading to difficulty walking, squatting or climbing stairs were asked to attend a clinical examination. Study physicians diagnosed hip OA based on history of diagnoses and on clinical evidence. Between 2000 and 2001, subjects from the original sample were identified and invited to a follow-up examination (n = 1,286). Of the 909 agreeing to participate, 69 were excluded for having a hip OA diagnosis at baseline or withdrawing from the workforce, leaving a final study population of 840. This represents only 11.6% of the original 7,217 participants, an extremely low follow-up rate. Non-participants, including those not invited and those declining participation, were older, had fewer years of education, higher BMI, and were less likely to be in a sedentary occupation and to have reported regular leisure-time physical exercise at baseline compared to members of the final study population. All of these factors are potentially related to hip OA, and the differences by participation status indicate that selection bias may have affected the results. If so, the results could be biased in either direction.

Hip OA was diagnosed at the follow-up examination using the same clinical criteria as were used at baseline. Occupational exposures, self-reported at baseline, were classified into 6 groups: Group 1, light sedentary work; Group 2, other sedentary work but involving fairly heavy objects; Group 3, light standing work or light work involving movement; Group 4, fairly light or medium heavy work involving movement; Group 5, heavy manual work; and Group 6, very heavy manual work. Logistic regression was used to compare odds of definite or probable hip OA among exposure groups, adjusted for age, sex, years of education, BMI, smoking status, alcohol intake, leisure time physical activity, and history of injury. Compared to those with light sedentary work, odds of hip OA was statistically significantly higher in Group 4 workers (OR = 3.1, 95% CI: 1.2, 8.0) and Group 5 workers (OR = 6.7, 95% CI: 2.3, 19.5). Only 12 people were classified in Group 6, and no hip OA cases were observed among them. Group 4 occupations were described as involving “a great deal of moving about and a fair amount of stooping down or carrying light objects, walking up and down the stairs or fairly rapid motion over rather long distances,” and group 5 work involves “either mostly standing work involving much lifting of light objects or lifting and carrying heavy objects, drilling excavating, hammering, etc., but with some sitting or standing.” Note that the categories combine multiple activities and do not offer any quantitative descriptions of weights lifted, distances walked, etc.

Thelin et al. (case ascertainment score: 2; exposure assessment score: 1) [61]) followed a cohort of 1,220 Swedish farmers, 1,130 non-farmer, but occupationally active, rural referents, and 1,087 urban referents from 1990 to 2003. Hip OA cases were determined through the Swedish national register of hospital care using ICD-9 and -10 diagnosis codes. Exposure information, apart from occupational category, was not collected. Hazard ratios for hip OA in farmers compared to both referent groups were calculated using Cox regression, with adjustment only for age. Farmers had three times the risk of hip OA (HR = 3.0, 95% CI: 1.7, 5.3) compared with the urban referent group; risks among the rural, non-farming group were similar to the urban referents (HR = 1.2, 95% CI: 0.8, 1.6).

Tuchsen et al. (case ascertainment score: 2; exposure assessment score: 1) [62]) used Danish national population and hospital registers to identify all employed men aged 20–59 years in 1981, 1986, 1991 and 1994. The men were followed for four years to identify first hospital admissions for hip OA, and standardized hospitalization rates (SHR) were calculated in a manner analogous to standardized mortality rates, using data for all employed men in Denmark. The SHR for hip OA was generally about twice as high as expected for farmers compared to the general population of Danish men, with some variability in the point estimates depending upon which occupational code was used to identify farmers. The lowest reported SHR was 114 (95% CI: 89, 147) for men “employed in agriculture and horticulture” in 1981–1985, and the highest was 286 (95% CI: 262 to 313) for “self employed farmers” in 1994–1999. The only exposure metric used in this study was occupational title, and no potential confounders were measured or assessed.

Thus, although these five cohort studies would be considered high quality based only on their design, they were each methodologically weak due to their choice of exposure metrics (job title or semi-quantitative categories) and/or the lack of control for confounding.

Two of the six studies that provided quantitative exposure data and met outcome ascertainment requirements were determined to be of low quality based on high likelihood for bias and confounding [63], [64], and one study was internally inconsistent in its exposure estimation methods [65]. All three were, therefore, excluded from the quantitative exposure-response analysis; study details are provided in the Appendix S1, and in Tables S1 and S2. The remaining three studies were judged to be of reasonable epidemiological quality and met our *a priori* requirements for characterization of exposure and outcome; they are described in detail, below:

### Croft 1992b (Case ascertainment score: 2; Exposure assessment score: 3) [66]

Croft, Cooper et al. performed a case-control study among men ages 60–75, identified from hospital x-ray registers as having undergone an outpatient urogram between 1982 and 1987, most often for prostate symptoms. Men who had a THR for hip OA or had JS ≤2.5 mm were identified as cases. A subset of “severe” cases included those with a THR for hip OA or JS ≤1.5 mm in at least one hip. Controls were those men identified by urogram review of having a JS ≥3.5 mm in both hips and no other radiographic indication of hip OA. The proportion of the cases identified by THR, and the JSN information for these cases, is not reported. While it seems unlikely that a THR for hip OA would occur without severe joint space narrowing, it is not clear from the methods whether cases identified by THR also met the minimal joint space criteria.

Lifetime occupational histories were determined through blinded interview, and then coded according to the 1970 Office of Population Censuses and Survey classification. Exposure levels were determined by length of time spent in specific work activities. Cases and controls had similar participation rates (69% and 68%, respectively), and a total of 245 (53 severe) cases and 294 controls completed interviews.

Associations between hip OA and duration of exposure to various work activities were examined using logistic regression, adjusting for age group and hospital. Height and weight, although measured at the time of interview, were not controlled in the analyses. Risk of severe hip OA in this study was found to be higher for those with the highest BMI, but the association was not statistically significant (OR for hip OA comparing highest vs. lowest tertiles of BMI was 1.6, 95% CI: 0.7, 3.4). Nevertheless, an analysis of the potential for confounding by height and weight was warranted, and the omission of control for these variables may affect the magnitude of the reported risk estimates.

Hip OA was evaluated for its association with each of the following occupational activities: sitting, standing, or bending for >2 hours/day; kneeling or squatting for >30 minutes/day; walking or walking over rough ground for >2 miles/day; running for >1 hour/day; climbing ladders; climbing >30 flights of stairs/day; manually lifting or moving weights >56 lbs; and driving for >4 hours/day. In analyses including all cases (JS ≤2.5 mm), no clear patterns emerged with any occupational activities and the only statistically significant association with hip OA was among men with 20 or more years' exposure to jobs requiring standing for >2 hrs per day, among whom odds of hip OA were nearly doubled compared to men not required to stand at work. A positive association was also reported between odds of severe hip OA (JS ≤1.5 mm) and the number of years in a job requiring standing for >2 hours/day. Compared to those with <20 years of standing >2 hours/day, the OR for severe hip OA was 1.5 (95% CI: 0.5, 4.8) for those with 20–39 years of exposure and 2.7 (95% CI: 1.0, 7.3) for those with 40+ years exposure.

Time spent in jobs requiring manual lifting or moving weights >56 lbs was also positively associated with odds of hip OA: compared to those with <1 year of exposure, odds were higher for men with 1–19 years (OR = 1.2, 95% CI: 0.5, 2.9) and ≥20 years (OR = 2.5, 95% CI: 1.1, 5.7) of this type of work. Other activities were generally associated with higher odds of severe hip OA comparing those with longer vs. shorter duration of exposure, but the trends were not consistent, results were not statistically significant and the magnitude of the associations was small, with ORs not exceeding 2. After adjustment for combined exposure to standing, walking, walking over rough ground, heavy lifting, participation in sports activities and BMI, the odds of severe hip OA was doubled (OR = 2.1, 95% CI: 0.7, 6.6) and odds were 60% higher (OR = 1.6, 95% CI: 0.5, 5.1) among men with more than 20 years' exposure to heavy lifting and more than 40 years' exposure to standing >2 hrs/day, compared to men <20 years exposure to heavy lifting and <50 years exposure to standing >2 hrs/day, respectively.

The main strength of this study was its use of urograms from local hospital registries to identify cases and controls, minimizing misclassification and ensuring that subjects were drawn from the same population pool. The similar participation rates for cases and controls also suggested that selection bias was unlikely. However, the extent and type of illness in the controls is not described, beyond the statement that most urograms were performed due to prostate symptoms. This study is considered to be of good methodological quality, and it provides data that potentially could be useful for a quantitative exposure-response analysis.

### Roach 1994 (Case ascertainment score: 3; Exposure assessment score: 3) [67]

In a case-control study of US Military Veterans attending a Veteran's outpatient clinic to obtain hip radiographs for diagnosis of hip pain or as follow up for THR, Roach et al. identified 212 cases of hip OA based on either Kellgren or Croft grade 3 or 4. Four hundred eighty-one men from the same clinic with outpatient IV pyelogram x-ray films on file that allowed for visualization of the hip joint were identified as controls, if measured JS>1.5 mm. All participants responded to self-administered questionnaires to identify those with >15 years employment in jobs with activities requiring heavy or light lifting, defined on the basis of specific work activities whose forces on the hip joint were estimated in mathematical models. Compression forces of more than twice body weight were considered “heavy”, and compression forces of less than body weight were considered “light”. All others were classified as “intermediate”, and excluded from analysis. After adjusting for prior cancer diagnosis (mostly bladder and prostate), obesity at age 40 and history of running for exercise, men who had been exposed to heavy work for at least 15 years had 2.5 times higher odds of hip OA than men with light work (95% CI: 1.5, 5.0), and this association was statistically significant.

This study was considered to be of good quality. Its strengths included internal validity stemming from the objective identification of both cases and controls from the same patient population; the methods used to estimate work load were substantiated from prior research; and the questionnaire was carefully designed to identify the critical elements of work history needed for the estimation of work load. The main limitations of this study were as follows:

- Its reliance on long-term recall of work history may have introduced recall bias, if the cases with hip OA were more likely to accurately report or to over-report the work load they experienced in the past. If recall bias played a role in this study, the association between prior work load and hip OA would have been exaggerated.
- Some of the men with heavy work load may have been misclassified as having had intermediate exposures. Because the intermediate exposure group was excluded, the only effect would have been to reduce the statistical power of the analysis to detect differences between groups.
- The study sample design led to its inability to assess tobacco smoking as a potential modifier of the effect of work load. Although data on smoking history were collected, the study was designed such that controls were more likely to have had a cancer diagnosis than cases. Because smoking is a risk factor for the types of cancer most commonly diagnosed among the controls, the controls were also more likely to have been smokers than the cases. Statistical control for confounding by both factors was thus necessary.

The authors categorized work load using different, incompatible mathematical models to estimate the compression forces conferred by various activities. They did not distinguish between quasi-static (average force) and dynamic peak forces.

### Vingard 1997b (Case ascertainment score: 3; Exposure assessment score: 3) [68]

This is a high-quality case-control study of women aged 50–70 years, residing in one of 5 counties in Sweden between 1991 and 1994. Cases of primary hip OA (n = 242) were identified from the Swedish national THR register, with diagnosis performed according to a standardized protocol including clinical and radiographic examinations. Women with other forms of arthritis and/or a history of leg injury, thought to be a risk factor for development of hip OA, were excluded. Controls (n = 298) were recruited from population registers covering the same regions in which the cases resided, and were matched on age (within one year) at the time of the survey. Surgery had taken place an average of 4.5 years (range: 0, 13) prior to participation. Potential controls with hip disorders were excluded.

Structured, in-person interviews were conducted with each woman to determine health and medication status and history, number of children, height and weight, and occupational and non-occupational physical activity history from age 16 until age 50. Categories of activities including hours spent sitting and standing, numbers of flights of stairs climbed, number of jumps, and number of heavy lifts were divided into categories of low (i.e., lowest 25%), medium (i.e., 25% to 75%) and high (75% to 100%) based on the distribution observed among controls. Information was collected and categorized separately on sports activities.

After adjustment for age, body mass index, use of cigarettes, past sports activities, number of children, and hormone therapy, high exposure to each of the work activities except for number of hours per day spent sitting was associated with higher odds of hip OA, with ORs between 1.5 and 2.1. High exposure to non-occupational physical activity was associated with a two-fold higher odds of hip OA (OR = 2.3, 95% CI: 1.5, 3.6), and sports activity increased the odds of hip OA when cross-classified by levels of occupational activity.

Strengths of this study included: its focus on exposures occurring prior to the surgery for cases (i.e., attention to temporal ordering of exposure and outcome); careful questionnaire design to identify activities contributing to physical load on the hip during periods of similar activity intensity; separate ascertainment of work and non-work activities; assessment and control of confounding, including tobacco use, hormone therapy, and BMI at age 40; and use of an enumerated population to develop the study population, minimizing selection bias and allowing for risk estimation. Selection bias is also minimized by the high and relatively similar participation rates reported for the cases (90%) and controls (82%).

The primary limitations of the study stem from its reliance on exposures recalled from the distant past (30–40 years prior) and the resulting possibility of potentially differential misclassification due to recall bias. For example, recall of recent activity levels may contaminate recall of activities from years ago. If this occurred, women with hip OA may have under-reported their past activity levels, because they would be less likely to have remained physically active compared to women without arthritis or knee problems. If this occurred, the magnitude of the observed associations would be attenuated.

When we compared exposure metrics across the three studies, we found that the use of biomechanical models and exposure measures differed so greatly that the data could not be combined. The individual quantitative results of the three studies are summarized in Tables 4 and 5. Overall, the data suggest risk of hip OA may increase with long term exposures to standing and heavy lifting at work, but the risk is not high and there is a large amount of variability in the results. There is some indication that long term occupational exposure to stair climbing may increase risk of hip OA, but results are inconsistent. Sitting at work does not appear to be associated with risk of hip OA based on these data.

**Table 4:** Standing, Sitting and Stair Climbing results from three studies eligible for exposure response analysis.

Published results		
Author, year	Odds ratio (95% confidence interval)	Exposure categories
Standing		
Vingard, 1997b	1.0 (referent)	<22,792 hours
	1.4 (0.8, 2.2)	22,793–51,546 hours
	1.6 (0.9, 2.8)	51,547–67,760 hours
Croft, 1992	1.0 (referent)	<20 years
	1.5 (0.5, 4.8)	20–39 years
	2.7 (1.0, 7.3)	≥40 years
Roach, 1994	1.0 (referent)	<15 years(mean 2.2 years)
	2.2	15–24 years(mean 19.8 years)
	3.0	25–34 years(mean 28.9 years)
	2.2	>34 years(mean 41.2 years)

Sitting		
Vingard, 1997b	1.0 (referent)	<13,563 hours
	0.8 (0.5, 1.2)	13,564–35,816 hours
	0.8 (0.4, 1.3)	35,817–65,384 hours
Croft, 1992	1.0 (referent)	<1 year
	1.0 (0.4, 2.2)	1–19 years
	0.8 (0.3, 1.7)	≥20 years

**Table 5:** Heavy lifting results from three studies eligible for exposure response analysis.

**Heavy lifts**		
Vingard, 1997b	1.0 (referent)	<20,328 lifts
	1.1 (0.7, 1.7)	20,329–44,088 lifts
	1.5 (0.9, 2.5)	44,089–95,040 lifts
Croft, 1992	1.0 (referent)	<1 year
	1.2 (0.5, 2.9)	1–19 years
	2.5 (1.1, 5.7)*	≥20 years
Roach, 1994	1.0 (referent)	<15 years(mean 2.2 years)
	2.2	15–24 years(mean 19.8 years)
	3.0	25–34 years(mean 28.9 years)
	2.2	>34 years(mean 41.2 years)

Stair climbing		
Vingard, 1997b	1.0 (referent)	<64,680 flights
	1.3 (0.8, 2.0)	64,681–564,960 flights
	2.1 (1.2, 3.6)*	564,961–3,353,400 flights
Croft, 1992	1.0 (referent)	<1 year
	1.2 (0.6, 2.5)	≥1 year

*Results are statistically significant at alpha level of 0.05.

## Discussion

The objective of this review was to determine whether the existing body of literature on work load and hip OA is sufficient for developing preventive exposure guidelines. Studies were first evaluated on exposure characterization, outcome definition, and methodological quality, and only studies meeting requirements for exposure and outcome definitions and determined to be of adequate epidemiological quality were compared on results and conclusions.

An assessment of study quality depends on the authors including a detailed description of methods. If the methods were not sufficiently described in an article selected for review, a study that might otherwise have been considered high quality would have been excluded. It should be noted that the evaluation of the studies in this review was performed with the objective of providing valid evidence in guiding the development of measures to prevent work-related hip OA, and this objective influenced our quality assessments.

Six studies were identified as meeting exposure characterization, outcome definition, and three of the six were of good epidemiological quality. Despite differences in study design and methods, and potential for biases and heterogeneity, the six studies were consistent in their results suggesting increasing exposure-response trends with increasing duration [63], [66] or intensity [64], [67], [68] of heavy lifting or heavy work load. The majority of the published reviews addressing the association between heavy work load and risk of hip OA also concluded there is positive and consistent evidence for a causal association [22]–[31]. Several reviews attempted to characterize the exposure-response relationship between heavy lifting and hip OA, and concluded that long-term, frequent lifting of 10–25 kg loads increased risk [24], [25], [28]. Results were mixed for other frequently evaluated occupational risk factors for work load and hip OA, especially sitting, standing, stair climbing and walking.

### Potential biases

Studies in the English or German languages were selected for review, which may have introduced some bias if high quality, quantitative data that failed to show an association between work load and risk of hip OA were available in other languages, but inappropriately excluded. Eight studies in languages other than German or English (specifically: Dutch, Italian, French, Swedish and Danish) were identified in preliminary searches, but not included in this review.

Any literature review is subject to publication bias. We did not attempt to identify or obtain unpublished data, as peer review was a preliminary and *a priori* criterion for inclusion. Several of the papers discussed in this review reported null findings, however, suggesting that bias towards publishing only positive findings was at least not universal.

All of the six key studies with quantitative exposure data used the case-control design, which is more susceptible than prospective studies to recall and selection biases. Specifically, several may have misclassified participants' exposures or outcomes, and we identified those with the potential for uncontrolled confounding in the preceding summaries and the Appendix S1. Although the five available cohort studies were less prone to bias due to their design, these studies also were limited by methodological weaknesses including selection and information biases, misclassification of exposure or outcome, and use of non-quantitative exposure measures.

Exposures based on self-reported work history through questionnaire or interview can be strongly affected by recall ability, especially when data are collected on exposures experienced over decades of work [69]–[71]. For example, Coggon (1998) included people up to 91 years of age and characterized physical work load experienced anywhere from 24 to 70 years prior to the interview [63]. Depending on the wording of interview or questionnaire items, it can be extremely difficult for respondents to provide reliable and complete quantitative information on timing, duration, frequency and intensity of specific work activities. In addition, those diagnosed with a condition suspected of being related to physically demanding work may more accurately remember or over-estimate historical work activity compared to individuals without such conditions, as they may have actively reflected on potential causes after learning of their diagnosis or beginning to experience symptoms. Such recall bias was likely in several of the included studies in which participants were asked to characterize occupational exposures from a time years to decades prior to the interview [63], [64], [66]–[68].

Because radiographically confirmed hip OA does not always produce symptoms, misclassification of outcome may have occurred in studies that failed to verify that the nominal control group was free from hip OA. In addition, the studies that identified hip OA cases from THR registries or waiting lists may have excluded mild or moderate hip OA cases [63], [64], [68], [72]. Misclassification of outcome could artificially reduce the risk estimates either if non-cases were mis-categorized as cases or if cases were mis-categorized as non-cases, but would not introduce bias if it occurred more or less equally across exposure groups.

Selection bias was minimized in several studies by appropriately selecting cases and controls from same source population [66]–[68]. However, differential participation rates by cases and controls in one study (Coggon 1998) [63] indicated selection bias was likely to have occurred. Selection bias and uncontrolled confounding may affect the results of a study in either direction.

All of the key studies controlled for potential confounding by age and sex, either through restriction of the study population, matching, or statistical adjustment. Other risk factors identified in studies of hip OA include BMI or obesity [25], smoking [73], and leisure-time activity [27]. Previous joint trauma, joint deformity, and certain disorders, such as dysplasia, may increase risk of OA in the affected joint [18]. Participation in certain sports may also increase risk of hip OA [18]. Four of the six studies with quantitative exposure data controlled for BMI or obesity [63], [65], [67], [68], while the remaining two studies investigated differences in BMI, but did not control for it in their analyses [64], [66]. Five studies controlled for or considered some measure of non-occupational physical activity or sports participation [64]–[68], and two controlled for smoking [65], [68]. All studies excluded or adjusted for some combination of previous joint trauma, malformations, or history of a disorder that may increase risk of OA [63]–[68].

### Heterogeneity

Common sources of heterogeneity between research studies, in general, include differences in study methods, distribution of risks in the source populations, and inclusion and exclusion criteria. Heterogeneity was prevalent in the studies included in this review. Some evaluated very specific populations, such as farmers [61], [74], construction workers [59] or ballet dancers [57], while others included mixed populations. The ages included also varied considerably (e.g. 20 to 59 years [62], 20 to 80 years [75] or 65 to 75 years [66]), and this contributed to lack of comparability across study results. Use of different case definitions, exposure classification methods, and time periods of exposure relative to diagnosis also may have affected the comparability of results across studies:

- When cases are identified from THR registries or waiting lists [63]–[65], [68], disease may be more severe than when cases are identified radiologically [66], [67]. Eligibility criteria for THR may differ by location, so heterogeneity may be present even when studies identify cases from the same types of sources. Participants with less severe hip OA may be misclassified as non-cases, potentially reducing the magnitude of associations.
- All six studies with quantitative exposure data relied on self-reported occupational history to determine exposure status, but they used different methods to classify exposures. For example, Croft [66] coded lifetime occupational history to a standard classification system, while Coggon [63] recorded work history only for jobs held longer than one year. The time period of exposure relative to the onset of hip OA varied across studies. Coggon [63] collected work history only up to age 30 or up to 10 years prior to study entry, while relevant occupational history for women participating in Vingard [68] covered time intervals anywhere from 0–20 years prior to study entry.

### Diagnostic criteria, methods

The diagnostic criteria described in this body of literature included versions with and without radiological imaging data, and some investigators identified cases based on pending or performed joint replacement therapy (THR), clinical findings or previous medical diagnosis. In a series of 227 Dutch patients, Bierma-Zeinstra et al. [76] found that agreement was poor between methods using only clinical findings and those combining clinical and radiological diagnostic methods (kappa 0,11), but high between two sets of criteria that both included radiological assessment (either smallest joint spaces or Kellgren scores). Regardless, a valid diagnosis considering the level of morphological damage can only be expected if radiological criteria were part of case ascertainment. All six of the studies with quantitative exposure data also used objective imaging criteria for diagnosing cases, and all except Roach [67] identified potential cases from THR registries. Nevertheless, there may be subtle differences in the severity of the cases included in each study, because the indication for THR may not be consistently defined or applied.

### Exposure assessment

In spite of the consistent, positive association between risk of hip OA and heavy lifting, variability across studies in exposure assessment methods and data collected contributed uncertainty to the conclusions that can be drawn from this literature review, as well as uncertainty in developing preventive measures. For example, exposures were assessed using simple yes/no questions [75], job titles (e.g., job at first medical examination) [59], longest held occupation [75], and comprehensive interviews on physical loads [63]) as well as combinations of these methods. Only six of 30 studies reviewed provided quantitative exposure data, and only three studies provided quantitative estimates of lifted weights that were similar enough to be combined. Unfortunately, the data required substantial manipulation and application of assumptions before they were similar enough to be combined; although we provide quantitative exposure-response estimates, these should be considered rough indicators of the risk of hip OA associated with lifting. No other exposure was reported in quantitative terms that could be combined, so it should not be assumed that lifting is the only occupational risk factor associated with a graded risk of hip OA.

The following sections provide a more specific evaluation of the various exposure assessment methods encountered in this body of literature, and discusses their utility for developing preventive guidelines.

#### Simple exposure parameters – levels 1 and 2

Studies that used qualitative or semi-quantitative exposure assessments (levels 1 and 2), i.e., job titles and time in those jobs (e. g. Lindberg and Danielsson 1984, Thelin 1990, Chitnavis 2000, Rossignol et al. 2003, Thelin and Holmberg 2007, Jarvholm et al. 2008 [2], [59], [61], [77]–[79]) do not provide sufficient information to support development of recommendations for preventive measures. This is because of variability in the actual exposures associated with any given job title or profession: each includes a range of different jobs and tasks, so conclusions about the specific work activities that increase risk cannot be reached.

#### Complex exposure parameters – level 3

The assessment of frequency and duration of specific work activities can be used to develop preventive guidelines, if they can be linked to a quantitative estimate of physical strain. Simple identification of job tasks, not modified by intensity levels (e.g., walking, standing or sitting) allows a rough quantitative estimate of exposure that depends only on duration. More detailed estimates of risk can be developed by including the time spent in specific work activities and repetitiveness of tasks per time unit during the work day or week. More complex jobs, like lifting and carrying of loads, require more detailed data to adequately assess risk. Ideally, exposure assessments should include, for example, the weight of the load, the distance carried and/or the height lifted, and the frequency and intensity of exposure to the task.

Although six studies captured quantitative exposure data in at least some detail, variability and uncertainty in the estimated exposures remains. For example, Jacobsen et al. [53] assigned daily lifting operations of 50×20 kg and 20×50 kg as equivalent, assuming that intensity and frequency confer equal stress on hip joints; hence a reduction of the weight per load only reduces stress if the frequency of loads does not in turn increase. Other authors also used categories that considered both weight and frequency [63], [64], [66], [80]. While quantitative exposure assessments such as these are more useful than qualitative or semi-quantitative exposure metrics, it is still necessary to be cautious in comparing and interpreting exposures across studies. Specifically, when authors report quantitative exposure estimates as cumulative exposure time, there is a high likelihood of misclassification due to grouping study participants with long duration, low exposure and short duration, high intensity exposure. This will lower the chances of identifying an association between work tasks and risk of hip OA, if one exists. Exposure metrics that account for both intensity and frequency of effort are better, but are still subject to misclassification if grouped into categories that are too wide for either frequency or intensity, or if any of the categories are open-ended. Finally, no individual-level exposure measurements were completed for these studies, nor would individual measurements be practical for large-scale epidemiological assessments of work load. Thus, even the most apparently objective exposure metrics only provide graded estimates of exposure. If such metrics are used to estimate an average intensity or frequency of exposure in a given time frame, it is necessary to apply stringent assumptions that might not be valid.

Self-reported exposures are more practical for epidemiological studies, and they may be at least partially validated by conducting plausibility checks, for example, by comparing recalled exposures to daily work output diaries or quantitative exposure databases. Overall, there is a need for objective assessment of specific occupational exposures that are not subject to recall and reporting issues [81], that are accurate and detailed, and that are capable of characterizing hip joint strain from load-bearing activities. Besides the demand for objective exposure assessment, identified occupational risk factors for hip OA should be examined for their biological plausibility, i.e. it should be determined whether or not there is an identifiable mechanism for hip joint damage associated with each job task that appears to increase the risk of hip OA. An exposure-response analysis that fulfils these validation and plausibility requirements can provide the evidence needed to develop specific prevention strategies that include occupational exposure limits.

#### Hazard, exposure-response characterization

Profound differences in the procedures for assessment and quantification of exposures in the best currently available studies make it impossible to pool data across studies, or to clearly characterize the type and levels of work load that increase risk for development of hip OA. The pattern of increasing risk of hip OA associated with heavy lifting is consistent with the conclusions summarized in the other systematic reviews, several of which attempted to quantify the exposure-response relationship between heavy lifting and hip OA. These concluded that long-term, frequent lifting of 10–25 kg loads was sufficient to increase risk [24], [25], [28]. Only one review has individually considered exposure to stair climbing and risk of hip OA; it concluded there was insufficient evidence for a causal association [24], [28]. No reviews concluded there is a specific risk of hip OA associated with standing or sitting exposures.

In summary, the only type of physical work load that has been quantified and for which there is adequate and consistent evidence of an association with osteoarthritis of the hip is heavy lifting over a long time period. While it seems to be unlikely that weights below 10 kg have any relevant effect on hip OA, it remains to be established the level at which cumulative long-term heavy lifting exposure does indeed lead to an increased risk. We concur with the conclusions presented in previous systematic reviews: the lack of quantitative exposure data and/or reliance on cross-sectional or case-control study designs is a weakness of the existing evidence base. The studies reviewed here generally are limited by a lack of objective exposure assessment.

### Conclusions

This review attempted to identify occupational risk factors for hip OA, which are generally difficult to measure and usually have no gold standard for assessment. Despite variability across studies in their methods, populations, exposure and outcome definitions and measures, there is consistent evidence that extended exposure to heavy physical work load can increase the risk for osteoarthritis of the hip. The studies that provided quantitative exposure data consistently reported positive, graded associations between development of hip OA and occupational tasks or jobs requiring lifting and possibly standing. The association between hip OA and stair climbing or jumping was not clear, and there appeared to be no association between hip OA and sitting or walking on the job. Even taking into account clear problems in exposure assessment as discussed above, the consistent 2 to 3 fold increased risk for osteoarthritis of the hip in those participant groups with the highest physical work load suggests some association which cannot be fully explained by bias or confounding. Thus, we would conclude that very high physical work load is likely to be an independent risk factor for osteoarthritis of the hip, although the available data do not allow this to be defined. There is some indication of an exposure-response relationship, but quantification of this relationship is not possible based on the current body of literature.

### Options for prevention

The inability to determine a threshold value for physical work load limits the development of specific preventive approaches. General recommendations for guidelines to help prevent hip OA can be developed from these data, as follows:

- Job activities with high physical work load should be minimized to the extent possible.
- Training and guidelines for appropriate ergonomic approaches to materials handling and other work activities should be made available and implemented.
- In workplaces where jobs require exposure to high work load, companies should promote preventive health measures for workers. Programs should focus on limiting exposure to extended standing etc. as well as providing physical training to prevent musculoskeletal diseases.
- Better work organisation may reduce unnecessary physical work load.
- Older employees and employees with hip disorders, hip deformity, former injuries or known intrinsic risks for THR should not be exposed to high physical work load.
- Tasks related to agricultural work should be specifically evaluated to identify the components that increase stresses on the hip joint.

### Recommendations for further research

Several gaps in the existing body of literature on occupational work load and hip OA have been identified that limit the ability of current research to inform detailed preventive measures. Future research could improve upon aspects of exposure assessment, diagnosis definition, and study design as follows:

#### Exposure assessment

- To facilitate objective assessment of physical work load exposures, industry-specific job-exposure-matrices should be developed based on objective measurements, homogenous exposure groups, and biomechanical models that are valid and reproducible. The matrices could be established using measurement data from work shift monitoring in combination with laboratory studies on task specific hip joint loading.
- Studies on occupational physical work load (e.g. lifting and carrying) should measure not only the level of exposure but also the frequency and duration of the exposure, with attention paid to heterogeneous activity demands within and between job titles.
- If questionnaires are used to collect exposure information, data should be obtained by a trained interviewer. Measures of plausibility should be introduced to detect under- and overestimations of exposure, and to assess the probability of recall bias. Questionnaire data should validated, to the extent possible, against objective measurements [82] or exposure databases.
- If physical work loads are expressed in terms of compressive joint forces or joint moments, static (-mean) levels and dynamic peaks must be distinguished.
- High occupational physical work load does not necessarily implicate high physical loading of the hip joint. Thus, consistent biomechanical models for assessing the loads on the hip joints with respect to the different occupational tasks have to be developed and applied.

#### Diagnosis

- The diagnosis of hip OA should always include clearly defined radiological features (osteophytes, joint space narrowing or K&L-score grade 2 and above). Consideration of alternative diagnostic criteria, e.g., those described by Altmann 1991 [6], is also recommended.
- Different clinical patterns of hip OA (unilateral, bilateral or generalized form with multi-joint involvement) and their relationship with physical work load should be clarified.

#### Study design

Hip OA is a chronic, insidious disease that tends to develop slowly over an extended period of time. The precise timing of disease onset is often unknown, and incidence can be difficult to accurately measure. These characteristics present challenges in designing epidemiological studies to study risk factors for hip OA, and are especially prohibitive for prospective cohort studies. To balance cost-effectiveness and study quality, use of the nested case-control design is recommended for future studies, in which cases and controls can be identified within a well-documented baseline population (such as a medical insurance population). In addition to work related exposures, the data on additional risk factors for OA should be collected and evaluated: age; sex; body mass index or weight; previous joint diseases (including congenital diseases like hip dysplasia or femoracetabular impingements) and injuries; OA of other joint groups; and sport and other non-occupational activities that may increase risk of hip OA.

## Supporting Information

Table S1 **Main conclusions of systematic reviews of hip OA and occupational factors, and supporting studies.**
(DOCX)

Table S2 **Studies considered highest quality in reviews that also characterized heavy lifting exposures.**
(DOCX)

Table S3 **Summary of quality assessment: Thirty occupational epidemiological papers discussing osteoarthritis of the hip, with best quality papers indicated in bold face type.**
(DOCX)

Table S4 **Study Details.**
(XLS)

Appendix S1 **Studies contributing to hazard identification.**
(DOCX)
