# Supplementary material for: Epidemiological Evidence for Work Load as a Risk Factor for Osteoarthritis of the Hip: A Systematic Review
Source: PLoS One. 2012 Feb 14;7(2):e31521. doi: 10.1371/journal.pone.0031521 (PMC3279372; doi:10.1371/journal.pone.0031521)
Supplement: Table S1 — Main conclusions of systematic reviews of hip OA and occupational factors, and supporting studies. (DOCX) [file pone.0031521.s001.docx]

|  | **Reviews and their conclusions** | | | | | | | | |  |
| --- | --- | --- | --- | --- | --- | --- | --- | --- | --- | --- |
| **Main supporting studies** | Maetzel 1997 | Bolm-Audorff 2000 | Lievense 2001 | Schouten 2002 | D’Souza 2005 | Vignon 2006 | Jensen 2006/ 2008 | Bierma-Zeinstra 2007^1^ | Aluoch 2009 | Da Costa 2010 |
|  | Positive association | Consistent evidence | Moderate evidence | Clear association | Suggestive evidence | High level of evidence | Moderate -strong evidence | Moderate - strong evidence | Strong relationship | Reasonable evidence |
| Axmacher 1993 |  | X |  |  |  |  |  |  |  |  |
| Axt 1960 |  | X |  |  |  |  |  |  |  |  |
| Coggon 1998 |  | X | X |  |  | X | X | X |  |  |
| Croft 1992 BMJ | X | X | X |  |  | X |  | X |  |  |
| Croft 1992 Scan | X | X | X |  | X | X | X | X |  |  |
| Cvijetic 1999 |  |  |  |  |  |  | X |  |  |  |
| Elsner 1995 |  | X |  |  |  |  |  |  |  |  |
| Flugsrud 2002 |  |  |  |  |  |  | X |  |  |  |
| Heliovaara 1993 |  |  |  |  |  | X |  |  |  |  |
| Jacobsen 2004 |  |  |  |  |  |  | X |  |  |  |
| Jacobsson 1987 | X | X | X |  |  |  | X | X |  |  |
| Kellgren 1985 |  | X |  |  |  |  |  |  |  |  |
| Lau 2000 |  |  |  | X | X | X | X |  | X | X |
| Lawrence 1966 |  | X |  |  |  |  |  |  |  |  |
| Lindberg 1984 | X |  |  |  |  |  |  | X |  |  |

**Table S1**. Main conclusions of systematic reviews of hip OA and occupational factors, and supporting studies

|  | **Reviews and their conclusions** | | | | | | | | | |
| --- | --- | --- | --- | --- | --- | --- | --- | --- | --- | --- |
| **Main supporting studies** | Maetzel 1997 | Bolm-Audorff 2000 | Lievense 2001 | Schouten 2002 | D’Souza 2005 | Vignon 2006 | Jensen 2006/ 2008 | Bierma-Zeinstra 2007^1^ | Aluoch 2009 | Da Costa 2010 |
|  | Positive association | Consistent evidence | Moderate evidence | Clear association | Suggestive evidence | High level of evidence | Moderate -strong evidence | Moderate - strong evidence | Strong relationship | Resonable evidence |
| Olsen 1994 |  |  |  |  |  | X | X |  |  |  |
| Roach 1994 | X |  |  |  | X | X | X | X |  |  |
| Rogers 2002 |  |  |  |  |  |  |  |  | X |  |
| Rossignol 2003 |  |  |  |  |  | X |  |  |  |  |
| Rossignol 2005 |  |  |  |  |  |  |  |  | X |  |
| Sandmark 2000 |  |  |  |  |  | X |  |  |  |  |
| Thelin 1990 | X | X | X |  |  |  |  | X |  |  |
| Thelin 1997 |  | X | X |  |  |  |  | X |  |  |
| Thelin 2004 |  |  |  |  |  | X |  |  | X |  |
| Tuchsen 2003 |  |  |  |  | X |  |  |  |  |  |
| Typpo 1985 | X |  |  |  |  |  | X | X |  |  |
| Vingard 1991 Int J Epi |  | X |  |  |  | X | X |  |  |  |
| Vingard 1991 Scan | X | X | X |  |  | X | X | X |  |  |
| Vingard 1992 |  | X |  |  |  |  | X |  |  |  |
| Vingard 1997b |  |  | X |  |  | X | X | X |  |  |
| Yoshimura 2000 |  |  | X | X |  | X | X | X | X | X |
| 1. Bierma-Zeinstra 2007 performed a systematic review of reviews and based conclusions on Lievense 2001 and Maetzel 1997. Therefore, primary studies in these two reviews were indicated as supporting studies for Bierma-Zeinstra 2007 conclusions. | | | | | | | | | | |
