# Supplementary material for: Epidemiological Evidence for Work Load as a Risk Factor for Osteoarthritis of the Hip: A Systematic Review
Source: PLoS One. 2012 Feb 14;7(2):e31521. doi: 10.1371/journal.pone.0031521 (PMC3279372; doi:10.1371/journal.pone.0031521)
Supplement: Table S2 — Studies considered highest quality in reviews that also characterized heavy lifting exposures. (DOCX) [file pone.0031521.s002.docx]

**Table S2**. Studies considered highest quality in reviews that also characterized heavy lifting exposures

| **Main supporting studies** | Bolm-Audorff 2000 | Lievense 2001 | Jensen 2006/ 2008 |
| --- | --- | --- | --- |
|  | Causal relationship, long-term and intensive heavy lifting | Moderate evidence, frequent lifting of weights ≥25 kg | Moderate to strong evidence, 10-20 years of lifting 10-25 kg |
| Coggon 1998 | X | X | X |
| Croft 1992b | X | X | X |
| Elsner 1995* | X |  |  |
| Flugsrud 2002 |  |  | X |
| Jacobsson 1987 | X | X |  |
| Vingard 1991b | X | X | X |
| Vingard 1997b |  | X |  |
| Yoshimura 2000 |  | X |  |
| *We disagree with the assessment of Elsner et al., 1995 as “high quality”, because the authors evaluated exposure with a series of “yes or no” questions that were not previously validated. | | | |
