# Supplementary material for: Epidemiological Evidence for Work Load as a Risk Factor for Osteoarthritis of the Hip: A Systematic Review
Source: PLoS One. 2012 Feb 14;7(2):e31521. doi: 10.1371/journal.pone.0031521 (PMC3279372; doi:10.1371/journal.pone.0031521)
Supplement: Table S3 — Summary of quality assessment: Thirty occupational epidemiological papers discussing osteoarthritis of the hip, with best quality papers indicated in bold face type. (DOCX) [file pone.0031521.s003.docx]

**Supplemental Table S3.** Summary of quality assessment: Thirty occupational epidemiological papers discussing osteoarthritis of the hip, with best quality papers indicated in **bold face type.**

| **First author, publication year** | **Study Design** | **Potential biases** | **Confounders, modifiers considered, controlled** |
| --- | --- | --- | --- |
| Axmacher and Lindberg, 1993 | Cross-sectional | Selection | Age, gender; no controlled analyses |
| Chitnavis, Sinsheimer, et al. 2000 | Cross-sectional | No valid comparison group | Ethnicity |
| **Coggon, Kellingray, et al. 1998^1^** | Case-control | Selection bias | Age, sex, source of medical care, BMI, history of hip injury, presence of Heberden’s nodes |
| Croft, Coggon, et al. 1992a | Cross-sectional | Selection | Age, height, weight |
| **Croft, Cooper, et al. 1992b^1^** | Case-control | Controlled | Age, hospital, duration of exposure |
| Cvijetic, kanic-Ozegovic, et al. 1999 | Cross-sectional | Information bias | Age, BMI, gender |
| Flugsrud, Nordsletten, et al. 2002 | Cohort | Controlled | Age, BMI, gender, weight, marital status, smoking status, leisure time physical activity |
| Heliovaara, Makela, et al. 1993 | Cross-sectional | Recall bias | Age, gender, BMI, previous injury |
| Jacobsen, Sonne-Holm, et al. 2004a | Cross-sectional | Cross-sectional analysis, results not presented | Age, sex, BMI |
| Jacobsson, Dalen, et al. 1987 | Cross-sectional | Recall bias | Uncontrolled |
| Jarvholm, From, et al. 2008 | Cohort | Controlled | Age, BMI |
| Juhakoski, Heliovaara, et al. 2009 | Cohort | Selection bias, large loss to follow-up | Age, sex, BMI,  smoking, education, alcohol, leisure activity, history of injury |
| Lau, Cooper, et al. 2000 | Case-control | Selection; recall; and information biases | Uncontrolled |
| Lindberg and Danielsson, 1984 | Cross-sectional | Cross-sectional analysis, results not presented | Uncontrolled |
| Riyazi, Rosendaal, et al. 2008 | Case-control | Controlled | Age, gender, geographic region, BMI, smoking status, menopausal status (women) |
| **Roach, Persky, et al. 1994^1^** | Case-control | Controlled | Age, gender, prior cancer diagnosis, obesity at age 40, history of running for exercise. |
| Rossignol, Leclerc, et al. 2003 | Cross-sectional | Unknown | Age, gender |
| Rossignol, Leclerc, et al. 2005 | Cross-sectional | Selection; information | Age, gender |
| Thelin, 1990 | Case-control | Unknown | Age, gender |
| Thelin, Jansson, et al. 1997 | Case-control | Unknown | Age, gender |
| Thelin, Vingard, et al. 2004 | Case-control | Controlled, but assessed farming-specific exposures, not standardized to be useful to general occupational exposures | Age, gender, residential area |
| Thelin and Holmberg, 2007 | Cohort | Controlled | Age |
| Tuchsen, Hannerz, et al. 2003 | Cohort | Controlled | Gender, age |
| Typpo, 1985 | Cross-sectional | Unknown | Age |
| van Dijk, Lim, et al. 1995 | Cross-sectional | Controlled | Gender |
| **Vingard, Hogstedt, et al. 1991b^1^** | Case-control | Controlled | Age, gender, current health, tobacco use, education, sports activities, height and weight |
| Vingard, Alfredsson, et al. 1991a | Cohort | Controlled | County, degree of urbanization, gender |
| Vingard, Lars, et al. 1992 | Case-control | Controlled; but assess sports only | Gender, age |
| **Vingard, Alfredsson, et al. 1997b^1^** | Case-control | Controlled | Gender, age, non-occupational physical activity, BMI, smoking, number of children, hormone therapy |
| **Yoshimura, Sasaki, et al. 2000^1^** | Case-control | Misclassification; incomplete reporting of results; recall bias | History of knee pain; education; BMI |

^1^Studies identified as potentially useful for an exposure-response analysis based on exposure and outcome characterization and methodological quality assessment
