# Supplementary material for: Epidemiological Evidence for Work Load as a Risk Factor for Osteoarthritis of the Hip: A Systematic Review
Source: PLoS One. 2012 Feb 14;7(2):e31521. doi: 10.1371/journal.pone.0031521 (PMC3279372; doi:10.1371/journal.pone.0031521)
Supplement: Appendix S1 — Studies contributing to hazard identification. (DOCX) [file pone.0031521.s005.docx]

**Appendix S1**

1. **Twenty-four studies contributing to hazard identification, but not quantitative exposure-response analysis**
2. Axmacher 1993 (Case ascertainment score: 2; Exposure assessment score: 1)^[[1]](#footnote-1)^ [61]

Hip OA prevalence in farmers was assessed in a cross-sectional study [61]. Members of the Swedish Farmers’ Safety and Preventive Health Association, a voluntary group consisting of about 20% of all Swedish farmers and farm workers, were asked either at an office visit or through a mailed questionnaire whether they had had bowel or urinary tract x-rays within the last 15 years. Farmers and farm workers who were not affiliated with the association were also approached, via postal inquiry. Those with radiographs available for review comprised the study population, and the prevalence of hip OA was compared with the prevalence of hip OA in the population of the city of Malmö. Methods for obtaining the hip OA prevalence in Malmö were documented separately [62].

Hip OA was defined as joint space (JS) <4 mm and/or structural changes, including osteosclerosis or cysts. These diagnostic criteria are non-specific and likely to overestimate the prevalence of hip OA. Farmers and farm workers were considered exposed, with no examination of specific work activities or duration of exposure. Prevalence of hip OA by age group and gender were presented for farmers and the general population, and the authors stated that the hip OA prevalence was statistically significantly higher in male farmers compared to male residents of Malmö based on chi-squared tests and Mantel-Haenszel rate ratios, but they did not report any numerical results.

This study was inadequate for an exposure-response analysis primarily because of the lack of information regarding specific work activities, quantification of intensity or duration of those activities, and failure to report quantitative results. Additional concerns included the use of prevalent cases and the non-specific diagnostic criteria for determining hip OA cases.

1. Chitnavis 2000 (Case ascertainment score: 3; Exposure assessment score: 1) [63]

Chitnavis et al. examined occupational information for 249 patients undergoing THR for hip OA and 125 patients undergoing total knee replacement (TKR) for knee OA among patients of a joint replacement center identified from medical records and radiographs. A total of 721 subjects were initially identified, and 319 were excluded, leaving 402 participants. Inclusion criteria were Caucasian ethnicity, only THR or TKR (not both) performed, and the “absence of a definite identifiable cause for OA in the joint(s) replaced.” Selection criteria were documented in another paper [64]. The main objective of the study was to compare risk factors in THR and TKR patients, and no other comparison group was included.

Exposure information was determined by interview, and occupational history was classified and scored. Occupational risks were classified only as white vs. blue-collar jobs, and summary scores were calculated as the product of occupational risk and years employed. Other work-related variables included the mean number of occupations, mean years employed, and the most common occupations.

Because there is no comparison group of non-cases and no description of classified occupational variables, results are not informative. The study was designed to compare risk factors for replacement of different joints (hip and knee) due to OA, and therefore results presented are not useful for elucidating exposure-response effects of work activities and hip OA.

1. Croft 1992a (Case ascertainment score: 2; Exposure assessment score: 1) [65]

In a cross-sectional survey, Croft, Coggon et al. assessed hip OA in 179 farmers and 71 office workers from rural communities in England. Subjects were identified through responses to a self-administered questionnaire, and a detailed occupational history was obtained by interview. Those reporting any work in agriculture were classified as farmers, and those who reported an entire career in office work were included in the referent group. Upon interview, 12 farmers were found to have less than 1 year in agriculture: these farmers were combined with the 71 office worker referents. No further exclusion criteria or work history details are reported for farmers or controls. The implicit assumption is that farming presents higher risk than office work for occupationally-related hip OA.

Hip x-rays taken within 6 months of the interview were reviewed: if none was available and subjects had not had a total hip replacement (THR), they were referred to a local hospital for x-rays of both hip joints. Hip OA was identified in 30 subjects by JS ≤1.5 mm in at least one hip or by THR surgery due to hip OA. Pre-operative radiographs were available for 15 of the 20 THR-identified cases, all of which showed JS ≤1.5 mm.

When participation rates were examined by self-reported hip pain, symptomatic farmers had a participation rate of 78% compared to 54% for asymptomatic farmers, 60% for symptomatic office workers, and 57% for asymptomatic office workers. The differing participation rates among farmers with and without symptoms related to hip OA indicates possible selection bias, which would have resulted in an over-estimate of the prevalence of hip OA in farmers.

Percentages of farmers with and without hip OA were calculated for various farm work activities, including: regularly driving a tractor for at least 3 months/year, reported by 61% of farmers with and 55% without hip OA; regularly driving a tractor for at least 4 hours/day, reported by 36% of farmers with and 30% without hip OA; and manually lifting or moving weights of 25 kg or more, reported by 96% of farmers with hip OA and 90% without hip OA. No statistical comparisons were made to formally assess differences. Odds ratios for hip OA prevalence by type and duration of farming were calculated using logistic regression analyses that adjusted for age in 2 year intervals, height in 3 strata, weight in 3 strata, and presence of Heberden’s nodes (hard or bony swellings in the finger or toe joints). Compared to referents, participants with 1-9 years in farming occupations had higher (but not statistically significant) odds of hip OA (OR=4.5, 95% CI: 0.8, 26.3). Those with 10 or more years of farming had a statistically significantly higher odds of hip OA (OR=9.3, 95% CI: 1.9, 44.5) compared to referents. No statistically significant associations with hip OA by type of farming were observed.

This study was not adequate for a quantitative exposure-response analysis of occupational risk factors for hip OA. It was considered to be “poor” quality due to its cross-sectional design, its reliance on self-reported exposure based on limited work history, and the potential for selection bias resulting in an over-representation of farmers with symptomatic hip OA in the study population.

1. Cvijetic 1999 (Case ascertainment score: 3; Exposure assessment score: 2) [66]

Cvijetic et al. obtained a random population sample of 678 adults aged ≥45 years from city records in Zagreb, Croatia. After excluding 88 subjects with rheumatoid arthritis or gout, 282 women and 298 men were included in the study population. Sampling methods and determination of arthritis and gout diagnoses in participants were not described, and no additional exclusion criteria were reported.

Occupational history was obtained via interview, and participants were classified into one of four exposure categories based on reported occupational physical demands: Category 1, mostly sedentary jobs with >80% of time spent sitting; Category 2, >80% of time standing; Category 3, >80% of time in non-sitting positions with low physical strain, including frequent walking and standing with lifting and carrying of objects up to 5 kg; Category 4, >80% of time in non-sitting positions with high physical strain, including frequent walking and standing with lifting and carrying objects over 5 kg. Participants were categorized by the interviewer and also asked to assign themselves to one of the exposure categories provided in a self-completed questionnaire. Self-determined and interviewer assigned exposure category classification did not coincide for 12% of participants. In these instances, participants “were asked to reconsider their choice and they agreed that the interviewer’s choice best described their occupation.” This may be an issue if interviewers were not blinded to the case status (not specified by the authors), and it introduces the potential for both information bias and misclassification of exposure.

Radiographs of the right hip were reviewed and graded according to the Kellgren scale, and those with a grade ≥2 were classified as hip OA cases. The number of hip OA cases was not reported, and those with hip OA only in the left hip may have been misclassified into the referent category.

Risk of hip OA by exposure category was analyzed using logistic regression with adjustment for age and BMI (body mass index). When odds of hip OA were compared between exposure categories using Category 1 as the referent, no statistically significant results were observed, and odds of hip OA were not higher in categories with higher intensity of occupational physical activity. Compared to those in Category 1, the highest odds ratios were observed for Category 2 (mostly standing) occupational exposures, with an OR of 1.50 (95% CI: 0.60, 3.21) for men and 1.45 (95% CI: 0.49, 3.58) for women.

Odds of hip OA were also examined by duration of exposure within each category; individuals with <20 years of exposure were defined as the referent group. Compared to women with <20 years of exposure, odds ratios were higher with longer duration of exposure in all categories, including Category 1 (mostly sedentary work); this suggests possible residual confounding by age, because age and duration of exposure are highly correlated. The only slightly statistically significant association was reported among Category 1 women with 20-29.9 years of exposure compared to those with <20 years, giving an odds ratio of 1.07 (95% CI: 1.03, 1.29). There was no pattern of increasing risk of hip OA by increasing exposure duration in men, nor were there differences in risk of hip OA across exposure categories in women, including those in sedentary occupations.

Problems with this study include the cross-sectional design, the potential for misclassification of both exposure and outcome and the lack of information on: identification and recruitment of the study population; participation rates; identification of exclusions; and the total number of hip OA cases. The extent to which any of these issues may have affected results is unknown: however, the combination of all issues causes low confidence in the accuracy and validity of results and leads to a poor quality rating.

1. Flugsrud 2002 (Case ascertainment score: 3; Exposure assessment score: 2) [67]

Flugsrud et al. matched exposure data from a large prospective cohort study and outcome data from a national THR registry to examine risk factors for hip OA in Norway. Between 1977 and 1983, the cohort study, conducted by the National Health Screening Service, enrolled 52,143 participants from 3 counties. Participants completed a self-administered questionnaire to provide information on occupational and leisure time activity levels. Follow-up for THR due to hip OA occurred from 1989 to 1998 using the Norwegian Arthroplasty Register. Cohort participants were matched to the registry via each person’s unique national identification code. Individuals were excluded if they did not attend the initial screening; if they had died or emigrated prior to the start of follow-up; and if they had already received a total hip replacement at the start of follow-up. After exclusions, the total study population included 50,034 participants. During follow-up, 672 participants underwent a first THR for primary hip OA and were identified as cases. By using THR surgery to define cases, some participants with hip OA who had not undergone THR may be misclassified as non-cases, potentially biasing risk estimates towards the null.

At baseline, participants classified their work during the past year as sedentary, moderate, intermediate, or intensive. Descriptions and examples of specific jobs that would fall into each category were provided. “Moderate” activity jobs included work with much walking (e.g., shop assistant, light industrial, education); “intermediate” activity work involved much walking and lifting (e.g., postman, heavy industrial, construction); and work with “intensive” activity was described as heavy manual labor (e.g., forestry, heavy farm work, heavy construction). This question is reported to be validated against both maximum oxygen uptake during exertion and activities recorded in a 7-day diary, but quantitative estimates of activity levels were not provided. There were 6-12 years between baseline assessments and start of follow-up, and the average duration of follow-up was nine years. Participants were categorized based on work activities performed in the year prior to the baseline assessment, i.e., 6-12 years prior to start of follow-up.

Analyses were performed using Cox proportional hazards regression analysis. Censoring occurred for emigration, death, end of follow-up, and THR performed for any reason other than primary hip OA. Analyses were adjusted for age at screening, BMI in quartiles, sex, body weight in quartiles, marital status, smoking status, and physical activity during leisure time in four categories.

Compared to those with sedentary jobs, risk of THR for hip OA was higher for higher categories of work intensity among both men and women. After adjusting for all variables described above, men had hazard ratios (HR) of 1.5 (95% CI: 1.0, 2.2) for moderate work, 1.7 (95% CI: 1.1, 2.4) for intermediate work, and 2.1 (95% CI: 1.5, 3.0) for intensive work compared to sedentary work. Compared to sedentary workers, women had HRs of 1.1 (95% 0.8, 1.6) for work was in the moderate category, 1.4 (95% CI: 0.9, 2.0) in the intermediate category, and 2.1 (95% CI: 1.3, 3.3) in the intensive category. Tests for trends were statistically significant at p<0.0001 for men and p<0.0003 for women. In comparison, after controlling for all other variables, risk was not associated with higher categories of intensity of leisure activity for either men or women.

The prospective study design limits the possibility of selection and recall bias and many important confounders are controlled in the analyses. However, the exposures analyzed by the authors were only based on qualitative descriptions and job title examples, and data were collected only for one year of work. It is not clear why occupational physical activity, but not leisure time physical activity, might be associated with hip OA. This study was rated moderate quality, and its score for the exposure assessment methods was not high enough for it to be included in a quantitative exposure-response analysis.

1. Heliovaara 1993 (Case ascertainment score: 2; Exposure assessment score: 2) [19]

A cross-sectional study of Finnish adults aged 30 and older was performed to examine potential risk factors for hip OA. Participants were systematically sampled from population registers of 40 areas to reflect the age and sex distributions of the Finnish population. A total of 8,000 people were initially identified, and 7,217 participated in a screening consisting of a self-completed questionnaire, structured interview for musculoskeletal health issues, and a joint function test. Subjects found to have any indication of musculoskeletal disease, including those with a positive disease history, symptoms, or suggestive findings, were invited to participate in the diagnostic phase: 3,775 subjects met at least one of these criteria, and 3,434 participated. Physicians diagnosed hip OA if either there was a documented history or there were definite findings in the physical status of one or both hips, based on the combination of recalled medical histories and medical records (including radiographs) provided by the participants. Of 2,016 women and 1,421 men who underwent the diagnostic examination, 232 women and 137 men were diagnosed with hip OA. The authors did not describe the criteria used to evaluate hip OA in the available radiographs, nor did they report the distribution of cases diagnosed based on symptoms versus radiological findings. However, it is stated that physicians were instructed to record a hip OA diagnosis in subjects with a symptom history indicative of OA, even in the absence of definite physical abnormalities. With diagnoses based on symptom history regardless of physical evidence and radiographs available only for an unspecified proportion of participants, it is likely that hip OA was over-diagnosed in this study population.

Occupational history was determined from the screening questionnaire. Exposures reported in the most current or longest held occupation were considered relevant. An exposure index of physical stress at work was calculated by the total number of exposures (range 0-5) to any of the following factors: lifting or carrying heavy objects; stooped or twisted work posture; vibration of whole body or use of vibrating equipment; continuously repeated series of movements; and working speed determined by a machine. The associations between these exposures and hip OA were evaluated using logistic regression, with adjustment for age, sex, BMI, and previous injury.

While the authors did report statistically significantly higher prevalence of hip OA among those in higher categories of physical stress at work, several methodological issues in study design and the exposure and outcome definitions make the results difficult to interpret and suggest the effect of bias. Hip OA prevalence was based on self-reported medical history and medical records, if any, provided by participants. The lack of reported diagnostic criteria and the stated preference for symptom history as a basis for diagnosis likely resulted in a misrepresentation of hip OA in the study population. The timing of disease or symptom onset with regard to relevant occupational exposures is not described: some cases had already been diagnosed with hip OA before participating in the study, making it possible that hip OA symptoms first occurred prior to documented exposures. The qualitative, broad exposure index categories are poor indicators for specific work activities, and render the exposure index not useful for elucidating the relationship between any of these specific activities and risk of hip OA.

1. Jacobsson 1987 (Case ascertainment score: 3; Exposure assessment score: 2) [68]

In a cross-sectional study performed in Sweden, occupational exposures were examined in 106 men with hip OA and 236 men with urological problems. The study population, identified from records from one hospital, consisted of 85 patients on the waiting list for THR due to hip OA and 262 patients with urograms for prostatic hyperplasia. Urograms were graded according to joint space narrowin (JSN), and 21 men with JS <3 mm were identified. These men, in addition to the 85 THR cases, made up the hip OA cases. There was no mention of radiographic review for THR cases.

Occupational history was provided via self-administered questionnaire. Work activities examined included “heavy lifting,” “much walking,” and “much standing”: however, these activities were not quantified and questions required only an ever/never response. The imprecise categories and lack of information on duration make these poor exposure indicators. Recall bias is a concern for any self-reported exposure and, in this study, especially for those hip OA cases waiting for THR: reported activity levels may reflect accommodations for current symptoms rather than an accurate picture of past activities, which are likely more relevant to the development of hip OA.

The numbers of cases and controls endorsing a positive history of the various occupational activities were compared using the chi-squared test, with no control for any confounders. Statistically significantly more cases than controls reported exposure to “heavy lifting” and “much tractor driving,” while no statistically significant differences between cases and controls were observed for “much walking” or “much standing.” Of interest, all THR-identified hip OA cases responded “yes” to the “heavy lifting” question. THR-identified hip OA cases also had the highest mean weight, while urogram-identified cases had the highest mean age, both of which are associated with hip OA.

This study is not useful for a quantitative exposure-response analysis because of the cross-sectional design, inadequate exposure definition, likelihood of recall bias, and lack of consideration of potential confounders.

1. Jacobsen 2004 (Case ascertainment score: 2; Exposure assessment score: 3) [69]

Jacobsen et al. investigated occupational risk factors among participants in the Osteoarthritis Substudy, a cohort within the Copenhagen City Heart Study (CCHS). Of 10,135 participants in the CCHS, 2,949 were identified for radiography of the pelvis based on positive responses to ≥4 musculoskeletal symptom questions on a self-administered questionnaire. Pelvic radiographs were also taken of 1,202 age and sex-matched controls with positive answers to ≤3 of the same questions. Reasons for exclusion were: pelvic inclination more than two standard deviations from mean (extreme values may cause errors in JS measurements); previous hip surgery; history of proximal femoral fractures; treatment of childhood hip disorders; or history of rheumatoid arthritis of any joint. The final study population consisted of 1,397 men and 2,289 women. Hip OA was defined primarily as JS ≤2.0 mm, with secondary radiographic indicators of maximum sclerosis ≥ mean + 2 SD; presence of osteophytes; or presence of subchondral cysts.

Occupational exposures since leaving school were self-reported by questionnaire in the CCHS. Each reported occupation was described as primarily seated, standing or walking with no repeated lifting, or daily repeated lifting. Occupations with daily repeated lifting were categorized based on frequency and intensity of lifting as follows: (1) 50 lifts x 20 kg or 20 lifts x 50 kg; (2) 50-100 lifts x 20 kg or 20-50 lifts x50 kg; (3) 100-250 lifts x 20 kg or 50-100 lifts x 50 kg; and (4) 250-500 lifts x 20 kg or 100-250 lifts x 50 kg. Using these categories, the authors reported no statistically significant relationships between radiographic features of hip OA and type and duration of occupational exposures. No specific results were presented, but the authors reported that odds ratios ranged from 0.7 to 1.0.

The major limitation of this study was its failure to report specific results. Daily lifting exposures were quantified based on intensity and duration, and the absence of associations with hip OA could be described as evidence for no exposure-response relationship. However, it is not clear how the referent group was defined for these comparisons, which hinders interpretation. Without documentation of the actual comparisons made, it is difficult to comment on the findings. Coupled with its cross-sectional design and the use of self-reported exposures, this study is of inadequate quality and detail for inclusion in an exposure-response analysis.

1. Jarvholm 2008 (Case ascertainment score: 3; Exposure assessment score: 1) [70]

Jarvholm et al. evaluated incidence of hip OA by job title in a cohort of 389,132 Swedish construction workers who received physical examinations every three to five years between 1971 and 1992. At each examination, participants’ job title, smoking habits, body weight and height were recorded. Job title from the first examination was used in the analyses. Hip OA incidence from 1987 to 1998 was determined through record linkage with the Swedish Hospital Discharge Register. Cases were defined as workers receiving surgical treatment for primary OA in the hip, and were identified by diagnosis codes (ICD 9: 715B; ICD 10: M16.0, M16.1) and surgery codes in the register. Cases classified as traumatic, with secondary OA, or with secondary hip replacement were excluded. Relative risks were estimated by Mantel-Haenszel statistics using white-collar workers in the cohort as the control group. Analyses were restricted to men ages 40-79 with BMI 17-35 kg/m^2^, and stratified for BMI at the first exam (4 categories) and 10-year age groups. After all exclusions were applied, the analytic cohort included 204,741 men, of whom 9,136 were considered to be white-collar workers. A total of 1,260 cases of hip OA were identified during the follow-up period. Relative risk estimates for hip OA by job title ranged from 0.77 to 1.58 when compared to white-collar workers, with none statistically significant.

The longitudinal design of the study, with repeated health examinations and linkage with a health outcome registry, are strong study design characteristics. However, there are several limitations. The category of white-collar workers, used as the comparison group, is not further described: it is assumed that these jobs represent mostly sedentary occupations, but examples of job titles are not provided. The number of hip OA cases is likely underestimated in the population, as only those severe enough to receive surgery were classified as cases. There are no descriptive statistics presented to indicate whether there were differences by job title in the distribution of age or BMI. If white collar workers in construction tended to be older than blue collar construction workers, they would have higher incidence of age-related hip OA and more time for the disease to progress to the point of needing surgery. If this was the case in the study population, it would lead to an overrepresentation of hip OA in the control group, which would dilute any true relationship between work activities and hip OA. The use of broad age categories (10-year age groups) creates the possibility of residual confounding by age. The use of job title at the first health examination may have resulted in some misclassification of exposure, or it may have accurately captured a relevant exposure period for cases by avoiding possible job changes due to early symptoms of hip OA. Job title alone, however, is not a quantitative exposure metric, and therefore is not useful for assessing exposure-response relationships.

1. Juhakoski 2009 (Case ascertainment score: 2; Exposure assessment score: 2) [71]

Between 1978 and 1980, Juhakoski et al. invited a representative sample of 8,000 Finns ages 30 and over, who were identified from population registers, to participate in the Mini-Finland Health Examination Survey. Ninety percent (7,217 subjects) completed baseline questionnaires, interviews, and laboratory and joint function tests. Those who reported experiencing hip pain leading to difficulty walking, squatting or climbing stairs were asked to attend a clinical examination. Physicians diagnosed hip OA based on history of diagnoses and clinical evidence, according to a standardized written protocol. Between 2000 and 2001, subjects from the original sample were identified and invited to a follow-up examination (n=1,286). Of the 909 agreeing to participate, 69 were excluded for having a hip OA diagnosis at baseline or withdrawing from the workforce, leaving a final study population of 840. This represents only 11.6% of the original 7,217 participants, an extremely low follow-up rate. The only reasons offered for not inviting many of the participants to the follow-up examination were death and not living in or around 5 major cities. Non-participants, including those not invited and those declining participation in follow-up examinations, were older, had fewer years of education, higher BMI, and were less likely to be in a sedentary occupation and to have regular physical exercise during leisure time at baseline compared to members of the final study population. All of these factors are potentially related to hip OA, and the differences by participation status indicate that selection bias may have played a role in the results.

Hip OA was diagnosed at the follow-up examination using the same clinical criteria as were used at the baseline examination. Both definite and probable hip OA diagnoses were included as cases. Occupational exposures, self-reported at baseline, were classified into 6 groups: Group 1, light sedentary work; Group 2, other sedentary work but involving fairly heavy objects; Group 3, light standing work or light work involving movement; Group 4, fairly light or medium heavy work involving movement; Group 5, heavy manual work; and Group 6, very heavy manual work. Example job activities were provided for each group to assist participants in classifying themselves. Logistic regression was used to calculate odds ratios for exposure groups, adjusted for age in years, sex, years of education in 3 categories, BMI in 3 categories, smoking status, alcohol intake, leisure time physical activity in 3 categories, and history of injury. Compared to those with light sedentary work, the odds of hip OA was statistically significantly higher in Group 4 workers (OR=3.1, 95% CI: 1.2, 8.0) and Group 5 workers (OR=6.7, 95% CI: 2.3, 19.5). Only 12 subjects were classified as Group 6 workers, and no hip OA cases were observed.

Compared to the baseline population, the final study population was on average younger, with a lower BMI and more likely to work in a sedentary occupation. These characteristics would tend to underestimate risk for hip OA: however, statistically significant associations with fairly light or medium-heavy work involving movement (group 4) and heavy manual work (group 5) were observed. Group 4 occupations are described as involving “a great deal of moving about and a fair amount of stooping down or carrying light objects...walking up and down the stairs or fairly rapid motion over rather long distances,” and group 5 work involves “either mostly standing work involving much lifting of light objects or lifting and carrying heavy objects, drilling excavating, hammering, etc., but with some sitting or standing.” The categories combine multiple activities and do not offer any quantitative descriptions of weights lifted, distances walked, etc. Therefore, their validity and reproducibility are unknown, and they are not informative for assessing quantitative exposure-response relationships. Additionally, the diagnosis of hip OA was based only on clinical indicators in the absence of previous history hip OA and cases included those with diagnoses considered either definite or probable. The likelihood of selection bias, misclassification of exposure and misclassification of outcome all contributed to the poor quality rating for this study.

1. Lau 2000 (Case ascertainment score: 3; Exposure assessment score: 3) [72]

This cross-sectional study was judged to be of poor quality. Data were collected between 1998 and 1999 from patients in Hong Kong hospitals. Cases comprised 30 men and 108 women with THR for primary hip OA sometime in the last 3 years who were currently either being treated or followed-up in the orthopedic units of regional hospitals. The diagnosis was radiographically confirmed and with a Kellgren grade of 3 or 4. Members of the comparison group were matched to the hip OA cases on age and sex, and were patients in government general practice clinics. Although the catchment areas for the study hospitals were the same, the authors did not comment on the comparability of patients of the two types of hospitals. If socioeconomic status differed between the patient groups, for example, then occupation and associated work load might also differ, leading to selection bias. Information bias may also play a role, because cases were objectively assessed for the presence or absence of hip OA by x-ray and physician diagnosed need for or prior THR, while members of the comparison group were identified based on self-reported absence of prior physician diagnosis and self-reported absence of hip pain. If some of the controls had undiagnosed hip OA, the association between hip OA and the risk factors analyzed would be reduced.

Cases and controls were interviewed to determine work history and job tasks, including: walking; squatting; kneeling; and climbing >15 flights of stairs/day for work. The only quantitative exposure measure used in the analysis and discussed in this report was based on responses to lifting of loads weighing > 10 kg and > 50 kg one to 10 times or more than 10 times per week. It appears from the case counts presented that the two exposure groups may overlap, i.e. those who reported lifting >50 kg may also have been categorized among those who lifted >10 kg, but this was not described by the authors.

Because the risks calculated separately for men and women were similar, only combined results are discussed here. The authors reported odds of hip OA associated with lifting > 10 kg one to 10 times per week (OR=1.01, 95% CI: 0.51, 2.0, based on 15 cases) and > 10 times per week (OR=3.17, 95% CI: 1.83, 5.52, based on 37 cases), but did not specify the referent category. Presumably, the comparison was against those whose work tasks did not include lifting >10 kg at least weekly. Odds of hip OA were higher for those with a history of lifting heavier weights at least weekly, but again the comparison group was not specified. For those lifting > 50 kg one to 10 times per week, OR=4.6 (95% CI: 2.0, 10.57; 15 cases) and for those lifting > 50 kg > 10 times per week, OR=3.39 (95% CI: 1.63, 7.04; 29 cases).

The major limitations of this study, leading to its “poor” quality rating, were its potential for information, selection, and recall bias, and its reliance on prevalent rather than incident cases - all of which are common in cross-sectional studies. Additional ambiguity in the results stems from the possible overlap between exposure groups and the uncertain identity of the comparison group used for the analyses. Finally, the authors failed to discuss or collect information on potentially confounding or modifying factors.

1. Lindberg 1984 (Case ascertainment score: 2; Exposure assessment score: 1) [73]

Lindberg and Danielsson studied the prevalence of hip OA in shipyard laborers, with “internal” controls including shipyard office workers and a sample of teachers, and “external” controls consisting of a random population sample from city records. Available radiographs from local hospitals and private clinics were searched for study participants, and hip OA was diagnosed by JS<4 mm in those age <70 and JS<3 mm in those age >70 years. Hip OA prevalence in the exposed and control groups was reported, but risk estimates were not calculated. Due to unquantified exposures, the cross-sectional study design and the lack of risk estimates, this study is not useful for assessing exposure-response associations.

1. Riyazi 2008 (Case ascertainment score: 3; Exposure assessment score: 1) [16]

This case-control study is nested within the GARP project (Genetics, Arthrosis and Progression), currently underway in the Netherlands, which aims to investigate the inter-relationships between genetic and external factors in the development of osteoarthritis. Cases comprised individuals with primary familial osteoarthritis of at least two sites. Hip OA was diagnosed on the basis of pain or stiffness on most days plus JSN evident on x-ray (criteria not specified) and with Kellgren score >2, or based on THR due to primary hip OA. Population controls were identified by random digit dialing procedures, and matched to cases on age (+ 5 years), gender and geographic region of residence. There were 382 cases, 93 with hip OA, and 345 controls included in the study. All cases and controls underwent clinical examination, including x-ray.

Standardized questionnaires were used to collect data on demographic characteristics, height and weight (to calculate BMI), smoking status, and, for women, menopausal status. Occupational histories were obtained, and job titles used to classify participants into categories of work demands based on loads lifted, and stooping, standing and walking requirements. The authors reported that the odds of familial hip OA was higher among those with physically demanding work (OR=3.3, 95% CI: 1.3, 8.2), however no more information on work load was provided.

Because of the lack of specific, quantitative information or analysis related to occupational physical demands, this study is not suitable for inclusion in a quantitative exposure-response analysis, in spite of its apparent methodological rigor.

1. Rossignol 2003 (Case ascertainment score: 2; Exposure assessment score: 1) [2]

Rossignol et al. requested physicians in France to refer two sequential patients presenting for OA of the hip, knee and/or hand during October and November 2000. Over 88% of the 10,412 patients who agreed to participate were seen for follow-up appointments. Diagnoses were confirmed by x-ray examination (criteria not specified) for 91.2% of cases, and by clinical symptoms for the rest. The authors compared the distribution of occupational category for all included cases, by age and gender, to the age-adjusted distribution expected from the French national census. For both men and women, the prevalence of OA (any location) was about twice as high as expected among patients in agricultural occupations.

Because of the use of prevalent cases, the lack of information on diagnostic criteria, the lack of information on potential confounders other than age and gender, and the use of qualitative exposure categories, this cross-sectional study is rated “poor” quality. It is not suitable for inclusion in a quantitative exposure-response analysis.

1. Rossignol 2005 (Case ascertainment score: 1; Exposure assessment score: 2) [74]

In a similar cross-sectional survey, Rossignol et al. had 1,394 French physicians recruit their first two patients consulting for hip, knee, or hand OA. The average duration of OA symptoms reported by the 2842 participating OA patients was more than seven years. All patients were between the ages of 20 and 80 and had to have held a job for at least one year; housewives and military personnel were excluded from the study. Physicians completed questionnaires on age, sex, height, weight, and medical history of patients. Occupational exposure to biomechanical stress was self-reported by patients to physicians as “yes” or “no” answers to five questions. Activities considered biomechanical stressors were: lifting or carrying heavy objects; uncomfortable position of the joint with OA; work with vibrating vehicle or tools; continuous repetitive movements; and working at a pace set by a machine. Prevalence rate ratios were calculated to compare reported exposures by site of OA, but no site-specific estimates of prevalence of stressors were provided. The longest-held occupation was self-reported and coded to one of 30 occupational categories, and expected rates of OA within each occupational category were derived from the French national distribution of occupations. These analyses indicated that the highest prevalence ratio for any OA was 2.9 (95% CI: 2.6, 3.3) for men who are or were self-employed in the construction, mechanics, clothing and food sectors, and 6.2 (95% CI: 4.6, 8.0) for women who are or were commercial and industrial cleaners.

Due to the use of prevalent cases of OA of various joints, lack of site-specific estimates of exposure prevalence and the limited, qualitative exposure definitions, this study is not useful for inclusion in a quantitative exposure-response analysis.

1. Thelin 1990 (Case ascertainment score: 3; Exposure assessment score: 1) [75]

In this population-based case-control study, Thelin compared occupation reported by 105 Swedish men who had had surgery for hip OA and 222 population controls. All participants were between 55 and 70 years of age. The author reported that farmers were overrepresented among cases (40.7%) compared to controls (25.2%), and that cases (48%) were more likely than controls (26%) to have been engaged in farming for at least 10 years. This study is rated “poor” due to its lack of quantitative exposure data and its failure to account for important confounders, especially BMI.

1. Thelin 1997 (Case ascertainment score: 3; Exposure assessment score: 2) [76]

In a second population-based case-control study, Thelin et al. identified men with previous radiological examinations and identified cases of hip arthrosis by joint spaces < 3 mm. Two male controls per case were identified from population registers, matched on birth year to the cases. Questionnaires were used to collect occupational history and other data from all cases and controls. The odds of being or having been in the farming industry for at least six months were 2.7 times higher (95% CI: 1.94, 3.7) for cases compared with controls, and cases with hip OA had more than four times the odds of having long-term (21 years or more) farm work compared to controls. Although differences in the risk of hip OA were associated with different farm tasks, the authors did not provide a quantitative assessment of physical work load for farm tasks they evaluated. This paper cannot contribute to a quantitative exposure-response analysis.

1. Thelin 2004 (Case ascertainment score: 3; Exposure assessment score: 2) [77]

A large proportion of farmers in Sweden (40%) belongs to and obtains medical care from the Swedish Farmers’ Safety and Preventive Health Association. Thelin et al. obtained medical records and x-rays from affiliated clinics, and identified men with THR for hip OA or JS *<* 3mm; these comprised the cases for the study. Controls were farmers also affiliated with the Swedish Farmers’ Safety and Preventive Health Association, and were individually matched to cases on age, sex and residential area [77]. Controls with symptoms or diagnoses of the knee or hip were excluded. Individual interviews, height and weight measurements (used to calculate BMI), lung function and blood samples were obtained for 369 cases and 369 controls. Interviews provided data on work history, work habits, family health history, sports activities, and personal characteristics including smoking habits up until the year when symptoms were first noticed (not diagnosed) and at ages 30, 40 and 50. After adjusting for potential confounding by other work activities, farmers who spent more than five hours per day caring for animals housed in barns had 13 times higher odds of hip OA (OR=13.3, 95% CI: 1.22, 145) than farmers with less animal care work or those who primarily raised crops.

Although this study was well designed and conducted and included quantitative, graded exposure estimates, its use of farming-specific exposures, while likely more accurate than estimated load weights on the joints, are not generally applicable to other occupations.

1. Thelin 2007 (Case ascertainment score: 2; Exposure assessment score: 1) [78]

Thelin et al. followed a cohort of 1,220 Swedish farmers, 1,130 non-farming (but occupationally active) rural referents, and 1,087 urban referents for hip OA from 1990 to 2003. Hip OA cases were determined through the Swedish national register of hospital care using ICD-9 and -10 diagnosis codes. Exposure information, apart from occupational category, was not collected. Hazard ratios for hip OA in farmers compared to both referent groups were calculated using Cox regression, with adjustment only for age. Farmers had three times the risk of hip OA (HR=3.0, 95% CI: 1.7, 5.3) compared with the urban referent group; the rural, non-farming group were similar to the urban referents (HR=1.2, 95% CI: 0.8, 1.6).

While the longitudinal study design is strong methodologically, no quantitative or semi-quantitative exposure measurements were reported. Additionally, important confounders were not controlled for. Therefore, this study is not useful for an exposure-response analysis.

1. Tuchsen 2003 (Case ascertainment score: 2; Exposure assessment score: 1) [79]

Tuchsen et al. used Danish national population and hospital registers to identify all employed men aged 20-59 years in 1981, 1986, 1991 and 1994. The men were followed for four years to identify first hospital admissions for hip OA, and standardized hospitalization rates (SHR) were calculated in a manner analogous to standardized mortality rates, using data for all employed men in Denmark. The SHR for hip OA was generally about twice as high as expected for farmers compared to the general population of Danish men, with some variability in the point estimates depending upon which occupational code was used to identify farmers. The lowest reported SHR was 114 (95% CI: 89, 147) for men “employed in agriculture and horticulture” in 1981-1985, and the highest was 286 (95% CI: 262 to 313) for “self employed farmers” in 1994-1999. The only exposure metric used in this study was occupation, and no potential confounders were measured or assessed. It is therefore of inadequate quality for a quantitative exposure-response analysis.

1. Typpö 1985 (Case ascertainment score: 3; Exposure assessment score: 1) [80]

Typpö examined 919 Finnish subjects with available radiographs for presence of hip OA. A total of 395 subjects were diagnosed as having hypertrophic, destructive, or mixed type OA: only destructive hip OA cases had a minimum JS requirement (<3 mm). Occupational history was determined via questionnaire, and exposures were classified as “mental” (i.e., white collar), light manual, medium manual, and heavy manual. No further qualitative or quantitative information on occupational exposure categories is described. Pearson’s chi-squared tests were used to compare hip OA cases to subjects without hip OA diagnosis by occupational category. Overall, there was a statistically significant difference in the distribution of occupational category for cases compared to controls: however, no risk estimates were provided, and it is unclear which specific categories differed. Additionally, the analyses compared all hip OA cases (including hypertrophic, destructive, and mixed type) to controls, and no analyses of occupation specific to destructive hip OA cases were performed. Due to the unclear exposure categories and the lack of risk estimates, this study was found to be inadequate for an exposure-response analysis.

1. van Dijk 1995 (Case ascertainment score: 3; Exposure assessment score: 1) [81]

Van Dijk et al. compared 19 retired professional ballet dancers aged 50 to 70 years with age, height and body weight-matched women attending plastic surgery and orthopedic surgery clinics for complaints not related to the lower extremities. All participants underwent radiographic screening to identify JSN, and several cases of hip OA were identified by this technique. The women in the comparison group had either not worked outside the home, or had had occupations with low physical demands. Except for classification by occupation, no exposure assessment was attempted, and the groups did not differ with respect to average joint spaces or presence of arthrosis. This study is not of adequate quality for a quantitative exposure-response analysis.

1. Vingard 1991a (Case ascertainment score: 3; Exposure assessment score: 1) [82]

Vingard et al. completed a study based in population registers in Sweden to determine the association between blue collar occupations, classified as having higher or lower than average exposure to static and dynamic forces on the leg, and risk of hospitalization for hip OA. Specific work tasks were classified according to estimated static and dynamic forces based on the results of biomechanical studies. The study population consisted of residents of 24 counties in Sweden, born between 1905 and 1945, who reported the same occupation on both the 1960 and 1970 census, and who responded to the 1980 census. The census data were linked to national hospital discharge registries to identify cases who had been hospitalized for OA of the knee or hip between 1981 and 1983. Exposures thus were acquired at least 10 years prior to diagnosis.

The association between risk of hip OA and work in occupations with high vs. low work load was adjusted for county of residence and degree of urbanization, and stratified by respondent gender and age (born 1905-1924 and born 1925-1945). Among men, the relative risks for hip OA were similar for the two age groups, 2.2 (95% CI: 1.6, 2.8) and 2.0 (95% CI: 1.6, 2.3) respectively. For women with a history of working in jobs with high vs. low work load, the relative risk of hip OA was 1.6 (95% CI: 0.9, 3.1) for those born 1905-1924 and 1.1 (95% CI: 0.9, 1.5) for those born 1925-1945.

Strengths of the study included its basis in population registers and its use of census data from 10 years prior to diagnosis to determine occupation. These factors indicate no information bias and collection of exposure data prior to disease onset, respectively. This exploratory analysis was rated as poor quality, however, due to the non-specific nature of the case definition (hospital treatment for hip OA, no diagnostic criteria described); the grouping of occupations according to subjective ratings; and the failure to account for potential confounding, especially for BMI.

1. Vingard 1992 (Case ascertainment score: 1; Exposure assessment score: 1) [83]

This study of the association between occupational physical demands, based on job title, and disability due to hip OA among men in Stockholm, Sweden was conducted in 1988. Participants were born between 1915 and 1934. Cases (n=140) had been granted disability retirement prior to age 65 on the basis of physician diagnosis of hip OA, and were identified from a national register of disability cases. Controls (n=298) were identified from among men in the general population, frequency matched to cases on age. All men with diagnosed psychiatric disorders, traumatic injury, rheumatic diseases or congenital musculoskeletal malformations were excluded.

Work histories were obtained from individual interviews; cases’ interviews focused on jobs held during the last 20 years of working life; controls were interviewed with respect to occupational history through 1981. Cases and controls who had worked for less than 20 years were excluded from the study. Accounting for work load experienced in the past is appropriate for a degenerative disease such as OA.

All occupations reported by cases and controls were categorized and scored according to work load on each of four body regions (neck and shoulders; low back; knee; and hip). The average work load score for each job and each body region was calculated, and cumulative exposure indexes were determined by multiplying the number of years in each job by the total work load rating, and dividing by the number of years worked. Scores of 1 were considered low; >1 to <2.5 were medium and scores >2.5 corresponded to high work load. These scores were then aggregated into a single score, rather than specifically determining the association between work load estimated for knee and hip, for example, and the risk of OA of those joints.

Descriptive analyses indicated that cases of hip OA disability retirement were more likely to report jobs with heavy aggregate work load scores (37%) than controls (8%), and less likely to report low aggregate work load scores than controls (14% vs. 49%). The relative risk of OA associated with moderate vs. low work load scores was 4.1 (95% CI: 2.4, 7.1), and the relative risk of hip OA associated with heavy vs. low work load scores was 12.4 (95% CI: 6.7, 23).

The main strengths of this study were its use of population registers, and therefore its good external validity, and its consideration of exposures experienced up to 20 years prior to retirement. Limitations, in addition to those noted above, included the poorly documented, potentially subjective diagnostic criterion (physician diagnosis) used for case finding and the semi-quantitative assessment of work load based on expert opinion, rather than estimated forces or weights or other, more objective, exposure metrics. Failure to assess or control for confounding, especially by BMI, is also a major consideration, and these limitations resulted in the study being rated as poor quality, and inadequate for inclusion in a quantitative exposure-response analysis.

1. **Three studies with quantitative exposure measures, excluded from quantitative exposure-response analysis due to their methodological limitations**
2. Coggon 1998 (Case ascertainment score: 3; Exposure assessment score: 3) [35]

A case-control study of occupational lifting and hip OA was performed in two health districts in England. Cases were identified from lists of patients waiting for THR due to hip OA, excluding those with a lower limb fracture in the past year, history of rheumatoid arthritis, ankylosing spondylitis, or other documented causes of secondary hip OA. Radiographs of all cases were reviewed and minimal joint space, overall Kellgren/Lawrence score, and other radiologic features of OA were recorded. Of all hip OA cases, 78% had JS≤1 mm and 96% had Kellgren/Lawrence grade ≥3. Controls were selected from a list of patients in the same general practice and matched to cases on sex and age (within 4 years), excluding those who had undergone hip surgery for OA and replacing those who declined to participate. These were considered general population controls, as almost everyone in England and Wales is registered with a general practitioner. Controls were asked about previous hip injury severe enough to cause consultation with a doctor, but no measures were taken to ensure that controls were free of hip OA apart from excluding those with previous THR.

Seven hundred and twenty-six cases and 1,060 controls were initially identified, and 611 case-control pairs were included in the final study population (participation rate of 84% for cases and 58% for controls). The authors explained that most exclusions were due to refusals to participate; the difference between cases and controls in participation suggests the possibility of selection bias, which would occur if those who refused differed from those who participated on some factor related to hip OA. There is not enough information available to determine the most likely direction of the effect on the risk estimates if selection bias did occur.

Occupational history, including intensity and duration of specific work activities associated with all jobs held longer than one year, was obtained through interview with a structured questionnaire. To reduce the possibility that occupational activities were modified due to early symptoms of hip OA, only job history up to age 30 or up to 10 years before entry into the study were considered for analyses. Reported jobs covered most of the working lives for men, but gaps in work history were common for women who left the work force to provide child care. The percentage of working lives reported in an occupation did not differ by case or control status (evaluated separately for men and women).

Exposures were considered “definite” when participants were confident that the work activity had occurred and “possible” when participants were unsure whether or not they had performed a specific activity. Analyses were performed only on definite exposures. Conditional logistic regression was used to assess relationships between hip OA and work activities, with adjustment for body mass index in three categories, history of hip injury, and the presence of Heberden’s nodes. The majority (94%) of controls was successfully matched to cases within 1 year of age, and all controls were within 3.4 years of cases’ age.

Men with a history of occupational lifting of ≥10 kg for at least 10 years before age 30 had about twice the odds of hip OA compared to men without heavy lifting exposure (OR=2.3, 95% CI: 1.2, 4.2). When heavier weights were evaluated according to duration of exposure, there was some indication of an exposure-response relationship: Men who lifted ≥25 kg for 5 to 9.9 years before age 30 had 60% higher odds of hip OA (OR=1.6, 95% CI: 0.7, 3.7), and OR=2.7 (95% CI: 1.4, 5.1) for men with exposures lasting ≥10 years compared to men without any exposure to heavy lifting. There was no difference in the odds of hip OA for men with 0.1 to 4.9 years of lifting ≥25 kg compared to men without occupational lifting exposures. Similar risks were observed for men lifting ≥25 kg up to 10 years before study entry, with a statistically significant increase in odds of hip OA for men with lifting exposures lasting more than 20 years (OR=2.3, 95% CI: 1.3, 4.4) compared to men with no exposure. No clear patterns for women were observed for lifting ≥10 or ≥25 kg up to age 30 or up to 10 years before study entry.

Risk of hip OA was also examined by intensity of lifting in those with at least 10 years of exposure. For exposures up to age 30, men lifting 10 to 24 kg had an OR of 1.7 (95% CI: 0.9, 3.4), those lifting 25 to 49 kg had an OR of 3.0 (95% CI: 1.5, 6.3), and those lifting ≥50 kg had an OR of 2.9 (95% CI: 1.3, 6.4) compared to men lifting <10 kg. A clear exposure-response pattern was observed in men when the same exposures were evaluated in those exposed up to 10 years before study entry. Compared to men lifting <10 kg, odds of hip OA increased monotonically with increased weights lifted, from 10 to 24 kg (OR=1.4, 95% CI: 0.7, 3.0), 25 to 49 kg (OR=1.9, 95% CI: 0.9, 3.9), and ≥50 kg (OR=3.2, 95% CI: 1.6, 6.5). Again, no patterns were observed in women for exposures experienced before age 30 or up to 10 years before the study.

Other occupational activities were explored by duration of exposure, with few informative or statistically significant results. In men, climbing more than 30 flights of stairs for 10 to 19.9 years yielded an odds ratio of 2.3 (95% CI: 1.1, 4.9) compared to those without stair climbing, however, no exposure-response trend was evident. Similarly, driving for more than 4 hours was associated with a statistically significantly increased risk for women with 0.1 to 9.9 years of exposure (OR=4.0, 95% CI: 1.2, 13.7) compared to none, but this result is based on only 4 exposed cases, and the OR was lower for women with >10 years of exposure. Exposures seemingly unrelated to odds of hip OA included sitting or standing for >2 hours, kneeling or squatting for >1 hour, and walking for more than 2 miles.

Strengths of this study include control for important confounders (age, BMI, presence of Heberden’s nodes, and previous hip injury), and analyses of both intensity and duration of heavy lifting. The authors also restricted the analysis to work activities occurring more than 10 years before study entry to mitigate the possibility that physical activity was reduced in response to early symptoms of OA. Secondary hip OA cases were, appropriately, excluded from the study. While hip OA cases were not identified based on JSN or Kellgren/Lawrence grades, the majority of cases met the criteria defined for both measures.

The main limitations of this study include the lack of radiographs to confirm disease-free status for controls, the potential for selection bias due to different participation rates for cases and controls, and potential recall bias for self-reported exposures. The failure to verify lack of hip OA among controls may have resulted in misclassification of some cases with less severe hip OA as controls. If this occurred, the observed associations would be biased towards the null; reported associations would only be of larger magnitude if there was no misclassification of controls. On the other hand, associations with exposures other than heavy lifting may have been masked. The potential for selection and recall biases is of greater concern, given the differences in participation rates for cases and controls, and self-report of exposures occurring up to 40 years or more in the past. Selection bias could influence risk estimates in either direction, while recall bias would typically inflate risk estimates. This study is appropriate for an exposure-response analysis, but must be regarded as lower quality due to the potential for biases to have affected the results.

1. Yoshimura et al., 2000 (Case ascertainment score: 3; Exposure assessment score: 3) [36]

Yoshimura et al. completed a case-control study in Japan comprised of 114 men 103 women aged 45 and older who had received or were waiting for THR due to primary hip OA. Among cases, 98.3% had Kellgren scores > 4, and 96.5% had JS < 1 mm. Controls were individually matched on age, sex, and residential district, drawn from population lists. The only exclusion criterion for controls specified by the authors was prior hip replacement due to hip OA. If this is accurate, then controls may have had mild to moderate hip OA, which would serve to artificially reduce the differences between cases and controls and would push the odds ratio towards the null value, 1.0.

Study participants completed a self-administered questionnaire to report all jobs since leaving school; work activities at main job; categories of weights lifted at least once per week at each job; and leisure time physical activities. The questionnaires were used to categorize participants according to whether or not they had been required to lift weights of at least 10, 25 or 50 kg at least weekly in either their first job or their main job. The authors did not specify whether or not these groups were mutually exclusive.

Odds ratios were calculated using conditional logistic regression, adjusted for history of knee pain and age at leaving school. Body mass index was also evaluated, and confounding was ruled out for this factor due its narrow distribution in the study population: the highest tertile for men was 23 kg/m^2^, and the highest tertile for women was 24 kg/m^2^.

The authors reported higher odds of hip OA associated with categories of weights lifted at both the first job and the main job. The referent group for these analyses was not specified; presumably, it was individuals who reported no or limited lifting at work. For those who lifted >10 kg at least once weekly at their first job, the adjusted OR for hip OA was 1.2 (95% CI: 0.6, 2.4). For those who lifted > 25 kg at least once weekly at their first job, the adjusted OR for hip OA was 3.5 (95% CI: 1.3, 9.7). No adjusted estimate for lifting >50 kg at first job was reported, probably because there were only 2 controls in this category. For analyses of lifting at the main job, odds ratios were 1.2 (95% CI: 0.6, 2.1), 1.5 (95% CI: 0.7, 3.0) and 4.1 (95% CI: 1.1, 1.52) for those lifting >10; >25; and >50 kg at least once per week, respectively. Again, the referent group was not specified, but it was probably individuals reporting no or limited lifting at work. When the authors separately analyzed unilateral and bilateral hip OA, they found a stronger association for unilateral disease than for bilateral disease for those whose first job required lifting >25 kg at least weekly (OR=13.2, (95% CI: 1.5, 111) unilateral hip OA; OR=1.5 (95% CI: 0.5, 1.3) bilateral OA). Similarly, for those who lifted > 50 kg at least weekly, the association was stronger for unilateral (OR=7.0, 95% CI 0.9, 58.2) than for bilateral disease (OR=1.8 (95% CI: 0.3, 11.6). Only unadjusted results were presented for unilateral and bilateral hip OA, probably due to small numbers of cases when thus stratified. Again, however, the referent group was not specified by the authors, making these comparisons difficult to interpret.

In addition to categories of lifting, several other potential occupational exposures were evaluated by Yoshimura et al., but these exposures were poorly characterized. Those whose first job entailed sitting > 2 hours per day had 40% lower odds of OA (adjusted OR=0.6, 95% CI: 0.3, 1.0) (referent group not specified). There were no associations observed between hip OA and standing; kneeling; squatting; driving; walking; or stair climbing, in the first or the main job.

This study provided quantitative exposure data and, thus, would have been useful for an exposure-response analysis, except for the following limitations:

- The potential for controls to have had some degree of hip OA;
- Potential recall bias stemming from self-reported work activities in the distant past; and
- The authors’ incomplete reporting of results (no referent group specified).

1. Vingard 1991b (Case ascertainment score: 3; Exposure assessment score: 3) [38]

This population-based case-control study identified 247 cases from among men aged 50-70 years treated in one of four referral hospitals in Stockholm, Sweden, for THR due to primary hip OA. Controls (322) were randomly sampled from the population of Stockholm served by the same hospitals. Men with congenital malformations, sequelae of polio, rickets or trauma were excluded from the study. Telephone interviews were conducted with all cases and controls to determine current health status, tobacco use, educational attainment, sports activities, occupational history and current height and weight, which were used to calculate BMI.

Data from the occupational history interview were used to categorize the men into high, low and intermediate categories of static and dynamic work load based on the cumulative frequency and duration of certain activities including standing, sitting, driving, climbing stairs and working in a twisted position. The frequency and amount of weight lifted on a regular basis was also quantified and categorized, based on the distribution of exposures reported by controls, into high, intermediate and low. The categories of exposures investigated were: Both static and dynamic forces on the leg; only static forces; only dynamic forces; total weight lifted (cumulative, in tons); number of lifts > 40 kg; and number of jumps between heights during working career. Exposures were analyzed from the start of work life through age 49, and separately for the periods through age 29, between age 29 and 39 years for ages 39 through 49.

After adjustment for age, smoking status, sports activities until age 29, and current BMI, odds ratios comparing the medium and high exposure groups to the low exposure group for each of the exposure categories analyzed (static forces only, dynamic forces only, etc.) were between about 2 and about 3, regardless of whether exposures were medium or heavy. Exposure to only static forces was associated with both the lowest risk estimate (moderate vs. low exposure OR=1.21, 95% CI: 0.64, 2.31) and the highest risk estimate (heavy vs. low exposure OR=2.92, 95% CI: 1.69, 5.05). The odds ratios reported for each type of exposure, comparing moderate and heavy exposure vs. low exposure, were generally of the same magnitude, but were more precisely estimated (i.e., had narrower confidence intervals) when exposures were limited to those experienced during younger ages (through age 29 and from 29 to 39 years). This suggests that exposures occurring within 10 years of diagnosis or treatment for severe hip OA are less pertinent to disease onset or progression than exposures experienced 10 or more years in the past.

In spite of limited information on the diagnostic criteria used to identify cases, this study was judged to be of high quality. Its strengths included assessment of and control for potential confounding, especially by age, BMI, smoking status and level of engagement in sports activities during youth. The authors evaluated the validity of the recalled data by including a second control group comprised of 335 men hospitalized for myocardial infarction. These men had a similar distribution of exposures as those reported by the general population controls. The authors provided detailed characterization of work activities, including separation into activities expected to confer static vs. dynamic forces; and they accounted appropriately for the timing of exposures to occupational and sports activities. The latter analysis allowed for the identification of a 10 year lag period prior to diagnosis and treatment for hip OA; exposures occurring during this interval added variability to the risk estimates without increasing the strength of the observed associations.

The major limitations of this study included its reliance on long-term recall for exposure data; the lack of historical information on BMI; and, as already mentioned, the lack of information on diagnostic protocols used to identify cases. The latter factor was mitigated by the authors’ focus on men with THR and thus, presumably, severe hip OA. This approach may have led to under ascertainment of cases, and the control group may have included men with mild or moderate hip OA. If so, the observed associations would be attenuated. It is unlikely that cases of severe hip OA were misclassified as controls.

1. The scoring system is described in Table 3 and Table 4 in the text. [↑](#footnote-ref-1)
